# Seismic Analysis and Design of Composite Shear Wall with Stiffened Steel Plate and Infilled Concrete

**DOI:** 10.3390/ma15010182

**Published:** 2021-12-27

**Authors:** Ke Wang, Wenyuan Zhang, Yong Chen, Yukun Ding

**Affiliations:** 1 Key Lab of Structures Dynamic Behavior and Control of the Ministry of Education, Harbin Institute of Technology, Harbin 150090, China; dingykun@sohu.com; 2 Key Lab of Smart Prevention and Mitigation of Civil Engineering Disasters of the Ministry of Industry and Information Technology, Harbin Institute of Technology, Harbin 150090, China; 3 China Northeast Architectural Design & Research Institute Co., Ltd., Shenyang 110006, China; dr_cy@126.com

**Keywords:** finite element analysis, mechanical mechanism, parametric study, design formulation

## Abstract

Several experiments are conducted to investigate the seismic behavior of composite shear walls because of their advantages compared to traditional reinforced concrete (RC) walls. However, the numerical studies are limited due to the complexities for the steel and concrete behaviors and their interaction. This paper presents a numerical study of composite shear walls with stiffened steel plates and infilled concrete (CWSC) using ABAQUS. The mechanical mechanisms of the web plate and concrete are studied. FE models are used to conduct parametric analysis to study the law of parameters on the seismic behaviour. The finite element (FE) model shows good agreement with the test results, including the hysteresis curves, failure phenomenon, ultimate strength, initial stiffness, and ductility. The web plate and concrete are the main components to resist lateral force. The web plate is found to contribute between 55% and 85% of the lateral force of wall. The corner of web plate mainly resists the vertical force, and the rest of web plate resists shear force. The concrete is separated into several columns by stiffened plates, each of which is independent and resisted vertical force. The wall thickness, steel ratio, and shear span ratio have the greatest influence on ultimate bearing capacity and elastic stiffness. The shear span ratio and axial compression ratio have the greatest influence on ductility. The test and analytical results are used to propose formulas to evaluate the ultimate strength capacity and stiffness of the composite shear wall under cyclic loading. The formulas could well predict the ultimate strength capacity reported in the literature.

## 1. Introduction

Steel–concrete composite shear walls are composed of web plates, stiffened plates, infilled concrete, and steel studs. The ultimate strength capacity and energy dissipation capacity of composite shear walls are higher than those of reinforced concrete (RC) walls. Researchers have been interested in the application of composite shear walls in buildings [1,2,3]. The relevant specifications for composite shear walls, such as seismic codes ASCE 7-10 [4] and AISC 341-10 [5], are formulated by permitting the use of composite steel plate shear wall (C-PSW) systems in earthquake zones. 

Researchers have conducted experimental studies on composite shear walls without boundary walls. Nie et al. [6], Mydin [7], Wright [8], Wang [9], and Nie [10] showed that a composite shear wall has high ultimate strength capacity and good ductility. The failure mode was local buckling of the web plate and fracture failure of the corner of the wall. The design formula for the width-to-thickness ratio of the steel plate was proposed. Zhang et al. [11] and Zhang et al. [12] showed that more channels could weaken the ultimate strength capacity and stiffness of the wall but could enhance the ductility and energy dissipation capacity of the wall. Increasing the axial compression ratio had little effect on the yielding bearing capacity and ultimate strength capacity of the wall but increased the capacity of stiffness degradation. Finally, formulas for calculating the ultimate strength capacity and initial stiffness were proposed. However, the formulas could not include all the relevant parameters, and the mechanical mechanism was not explicit due to the limitation of the test. Thus, numerical analysis of the composite shear wall is necessary.

Researchers have studied FE models and proposed design formulas for composite shear walls. Nguyen et al. [13], Epackachi et al. [14], and Rafiei et al. [15] established FE models and verified their accuracy. The damage process of infilled concrete under a cyclic load and the proportion of the contribution of steel to the total shear force were analysed. Parameter analysis of the connector in the wall showed that more connectors could improve the bearing capacity of the steel plate and that changing the spacing of the connector could affect the failure mode of the steel plate. Wei et al. [16] studied the axial compression performance of composite shear walls. The influence of distance-to-thickness ratios on the failure mode was studied, and a formula for the axial compression capacity of a composite shear wall was proposed. The higher axial compression ratio of wall [17,18] is beneficial to restrain the internal concrete and improve the compressive strength of concrete, so the energy dissipation capacity of composite shear wall is enhanced. Increasing the thickness of the steel plate [19,20,21,22,23] can increase the stiffness and ultimate bearing capacity of the wall, as the hysteretic curve of the wall is plumper. Varma et al. [24] simulated shear walls with different aspect ratios. When the aspect ratio was between 0.6 and 3.0, the coupling effect of the moment and shear force was obvious. Specifications [25,26,27] define the formula for the shear capacity of composite shear walls. The formulas for the shear bearing capacity and the flexural bearing capacity were given, but the formula for the flexural-shear coupling was not provided.

In summary, the seismic performance of the CWSC is usually affected by factors such as the axial compression ratio and the shear span ratio. The scholars have carried out the relevant experimental studies on the composite shear wall, laying a foundation for the research on the seismic performance of the wall. The ultimate bearing capacity and lateral stiffness of composite shear walls are important parameters of seismic performance. However, most of the relevant researches are qualitative studies, and the influence of parameters on the ultimate bearing capacity and lateral stiffness are not quantitatively analysed. Although some studies have obtained the formulas for the ultimate bearing capacity and lateral stiffness, they are all based on the test results. The predictive effect with other cross-sectional forms are unknown. In addition, the formulas do not consider the influence factors of the number of channels, so it is not comprehensive enough. 

**Figure 1:**
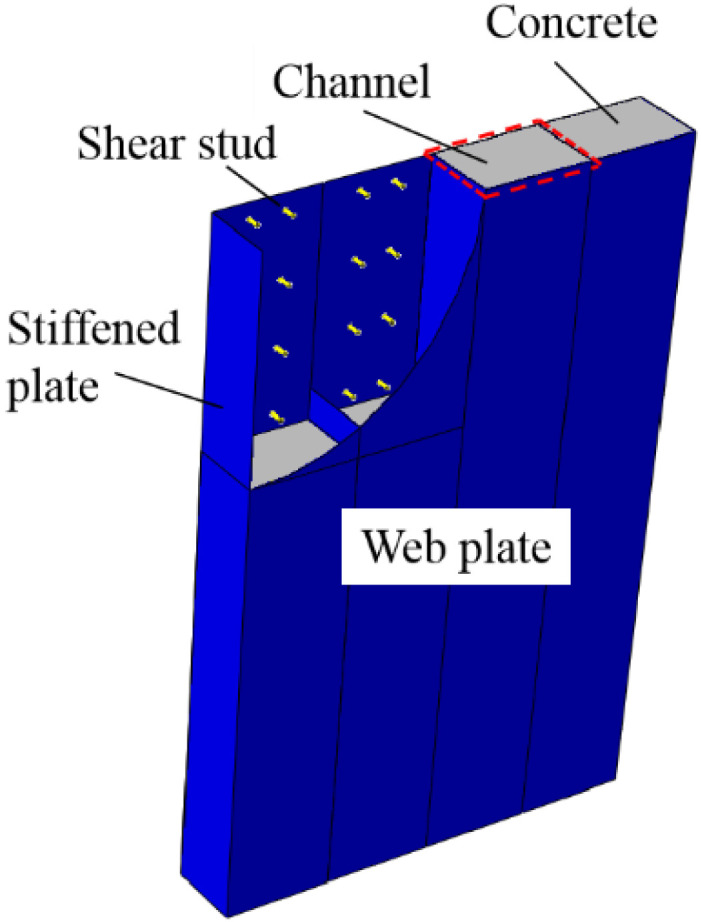
Composite shear wall in this paper.

In this paper, FE models are established with ABAQUS to simulate the seismic behaviour of composite shear walls under the cyclic loading and validated by tests. Figure 1 is a schematic diagram of composite shear wall. The mechanical mechanism, stress distribution and failure modes of the composite shear wall are researched by finite element analysis. A comprehensive parametric study is carried out to investigate the influence of parameters, including wall thickness, steel thickness, and shear span ratio, on the seismic behaviour. Formulas are proposed to predict the ultimate strength capacity and stiffness of composite shear walls and are validated by tests and parametric analysis. In addition, the other forms of composite shear walls are fitted by the formulas. 

## 2. Experimental Work

### 2.1. Sample Design

**Figure 2:**
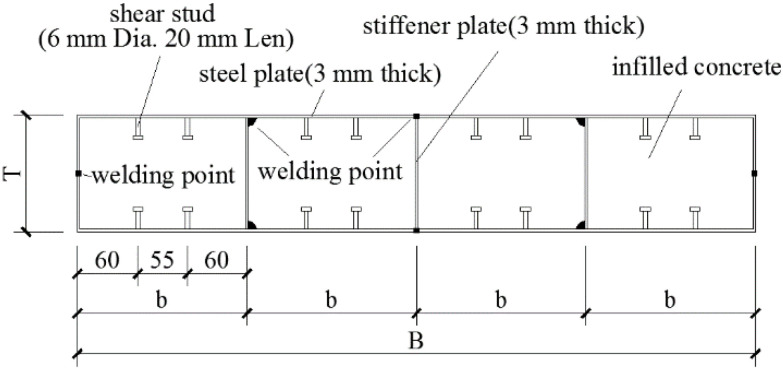
Details of the CWSC-1.

The author conducted experimental research on composite shear walls [12]. The steel plates were extended into the RC beam to prevent the samples from pulling out. The CWSC samples were constructed using double steel plates as the external component. The concrete was constructed as the infilled component. This experiment was based on the study of a shear wall in a super high-rise building [3], and five types of specimens were designed at a 1:5 scale. The CWSC samples were divided into several channels by the stiffened plates, as shown in Figure 2. The parameters of the samples are listed in Table 1. All the samples had a height of 1050 mm and a rectangular cross-section width of 700 mm. The thickness of the steel plate was 3 mm. The shear span ratio of all the samples was 0.75. The diameter of the stud was 6 mm, and the height of the stud was 20 mm.

### 2.2. Loading Programme and Test Setup

All the samples had the same axial force ratio (where *n* = 0.5), which was defined as Equation (1) [4].

(1) $$n=\frac{1.25N}{f_{c}A_{c}/1.4+f_{y}A_{s}/1.11}$$

where *f*_c_ is the axial compressive strength of the concrete; *f*_y_ is the yield strength of the steel plates; and *A*_c_ and *A*_s_ are the cross-sectional areas of the concrete and steel plate, respectively. 

**Figure 3:**
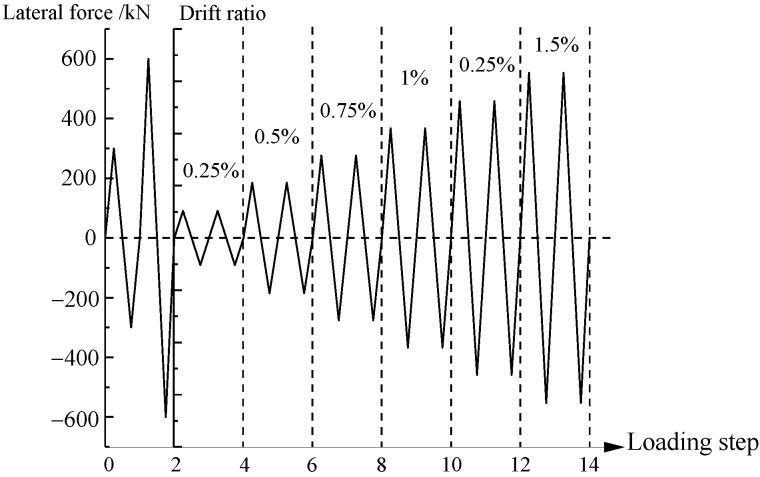
Loading history of test.

Horizontal loading was controlled by the force and displacement [28]. In the force-loading phase, the horizontal forces were 300 kN and 600 kN, and loading was performed in one cycle. Horizontal loading was changed to displacement loading when the drift ratio reached 0.25%. The displacement increment was 2.625/0.25% (in mm/drift), and loading occurred in two cycles. The displacement increment was increased to 5.25/0.5% (in mm/drift) when the test displacement reached 21/2% (in mm/drift). The test was stopped either when the horizontal force dropped below 85% of the maximum strength or when a constant vertical axial force could not be maintained. The loading history is illustrated in Figure 3.

**Figure 4:**
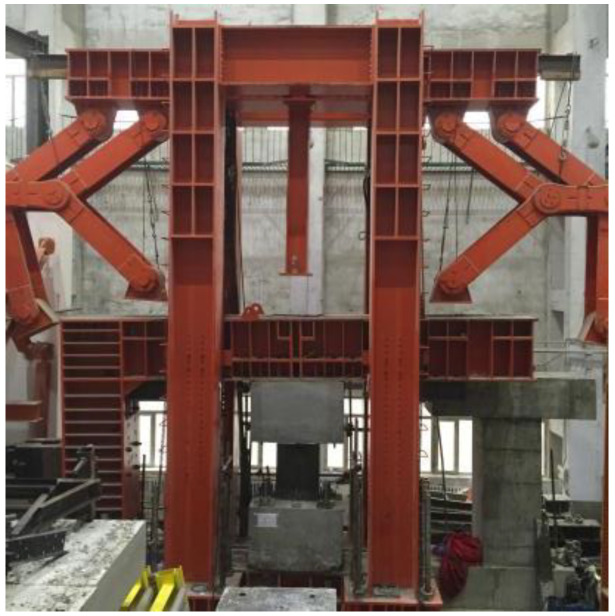
Test setup.

The test setup is shown in Figure 4. The specimen was laid between the top steel L-beam and the bottom steel beam, and the RC beams were fixed to the bottom steel beam and the top steel L-beam. The bottom steel beam was anchored to the ground, and the top steel L-beam was connected by three actuators, one in the horizontal direction and the others in the vertical direction. On the top steel beam, four supports were arranged to prevent the out-of-plane deformation of the specimen during the test. 

## 3. FE Modelling

### 3.1. Model Overview

#### 3.1.1. Part and Element of the FE Model

The FE model is composed of five parts, including the outer steel plate, steel studs, infilled concrete, concrete beams and reinforced cages. The outer steel plate is composed of a web plate and stiffened plate. 

**Figure 5:**
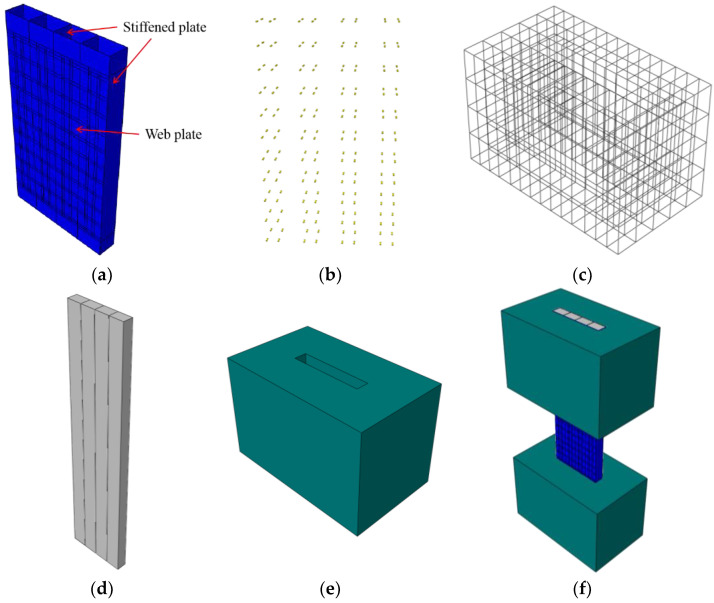
Element types of the FE model. (**a**) Outer steel plate; (**b**) Steel studs; (**c**) Reinforced cage; (**d**) Concrete; (**e**) Concrete beams; (**f**) Assembled FE model.

In order to simplify the calculation, the simple element type should be selected as far as possible. Since the thickness of the outer steel plate is less than 1/10 of its length and width, the stress change in the thickness direction can be ignored. So, the outer steel plate uses shell element (S4R). The change of stress in the thickness direction cannot be ignored in steel stud and concrete, because the sizes in three directions have little difference. The steel studs, infilled concrete and concrete beam use the solid element (C3D8R). The reinforced cage uses a beam element (T3D2), as shown in Figure 5. The assembled FE model is shown in Figure 5f.

#### 3.1.2. Contact of FE Model

**Figure 6:**
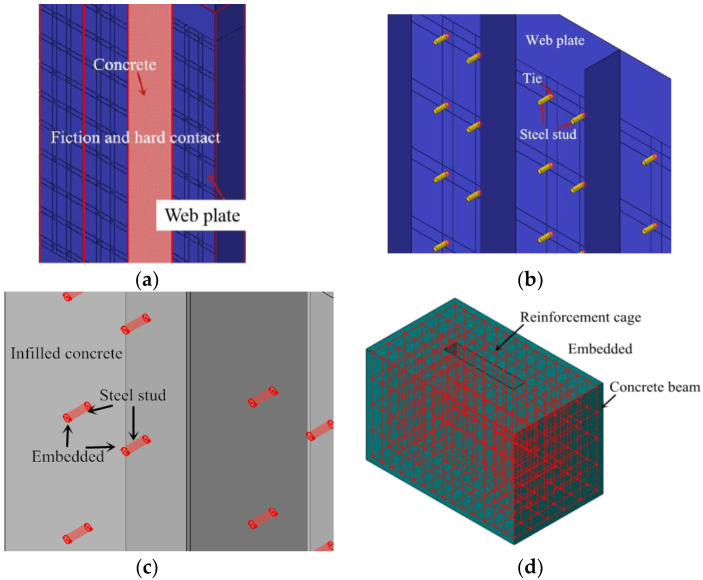
Contact between different elements. (**a**) Steel plate-infilled concrete; (**b**) Steel stud-web plate; (**c**) Steel stud-infilled concrete; (**d**) Reinforcement cage-concrete beam.

In the FE model, the friction contact mode is used between the steel plate and concrete. For the normal contact behaviour, the “constraint enforcement method” in the default option is chosen, and “hard contact” in pressure-over closure is considered. For the tangential behaviour, the penalty in the friction formulation is chosen, and isotropic directionality is considered. The tangential friction coefficient is 0.6 [29], as shown in Figure 6a.

In the test, the steel studs were welded on the web plate. Thus, the steel studs are tied on the steel plate in the FE model, as shown in Figure 6b. 

In the test, the reinforcement cage and the steel studs were poured with concrete. The reinforcement cage and the steel studs were fixed in the concrete. Thus, the reinforcement cage and the steel studs are embedded in the concrete beam and infilled concrete respectively, as shown in Figure 6c,d.

#### 3.1.3. Boundary Conditions

**Figure 7:**
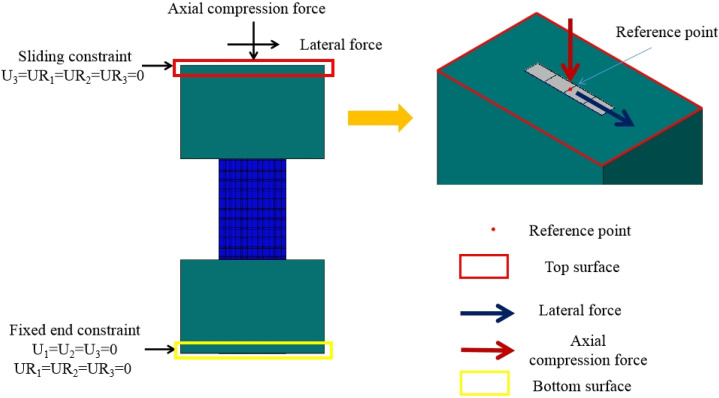
Boundary condition of the FE model.

In the test, the bottom beam was fixed to the ground through four bolts. The top beam is connected to the L-shaped beam through ten bolts, which limits the angle of the top beam and bottom beam. The boundary condition of the bottom beam is a fixed end constraint, and the boundary condition of the top beam is a sliding constraint, as shown in Figure 7. Therefore, six degrees of freedom are constrained at the bottom beam (i.e., U1 = U2 = U3 = UR1 = UR2 = UR3 = 0), and four degrees of freedom are constrained at the top beam (i.e., U3 = UR1 = UR2 = UR3 = 0).

A reference point is set on the top of the model, which is coupled with the top surface. The axial compression force and lateral force are applied on the reference point. The loading process used in the model is the same as the tests, as shown in Figure 7.

### 3.2. Steel Constitutive Model

**Figure 8:**
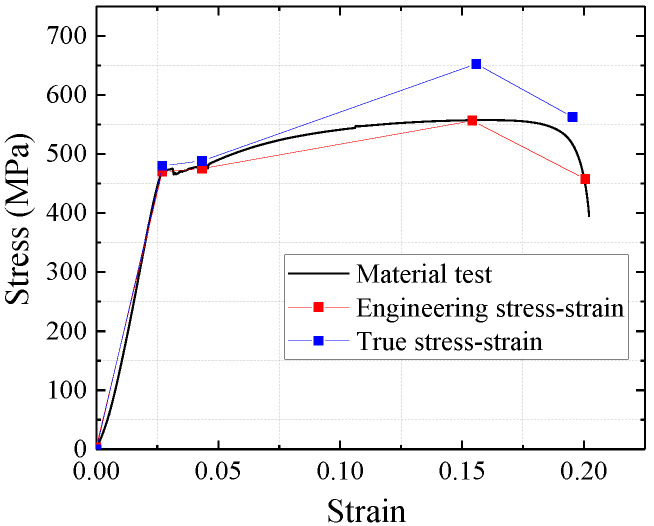
Steel constitutive curve.

The constitutive model of steel is a fourfold linear isotropic strengthening model. The constitutive curve uses the uniaxial stress–strain curve of the test and converts to the true stress–strain curve [13]. The constitutive curve of the steel is shown in Figure 8.

In the test process, the expansion of microcracks in the steel would cause a decrease in the ultimate strength capacity and stiffness of the material. Thus, the constitutive model of the steel must be considered the plastic damage.

The Wierzbicki [30] damage criterion considered that the excessive accumulation of plastic damage strain was the main cause of steel strength and stiffness degradation. When the equivalent plastic strain $\varepsilon^{{}^{{}_{\mathrm{pl}}}}$ of the steel exceeds the equivalent plastic damage strain $\varepsilon_{{}^{0}}^{{}^{{}_{\mathrm{pl}}}}$, the internal steel is fractured and starts to damage. Wierzbicki [30] and Cook [31] proposed a steel plastic damage model and considered the equivalent plastic strain $\varepsilon_{{}^{0}}^{{}^{{}_{\mathrm{pl}}}}$ to be associated with the degree of the triaxial stress η of steel. The degree of the triaxial stress η is defined as shown in Equations Equations (2)–(4).

(2) $$\eta =\sigma_{m}/\bar{\sigma }$$

(3) $$\sigma_{m}=\frac{1}{3}(\sigma_{1}+\sigma_{2}+\sigma_{3})$$

(4) $$\sigma_{\mathrm{Mises}}=\sqrt{\frac{1}{2}\left[{\left(\sigma_{1}-\sigma_{2}\right)}^{2}+{\left(\sigma_{2}-\sigma_{3}\right)}^{2}+{\left(\sigma_{3}-\sigma_{1}\right)}^{2}\right]}$$

where *σ*_m_ and *σ*_Mises_ represent the hydrostatic pressure and von Mises stress of steel, respectively. *σ*_1_, *σ*_2_, and *σ*_3_ represent the principal stresses of steel in three directions. 

**Figure 9:**
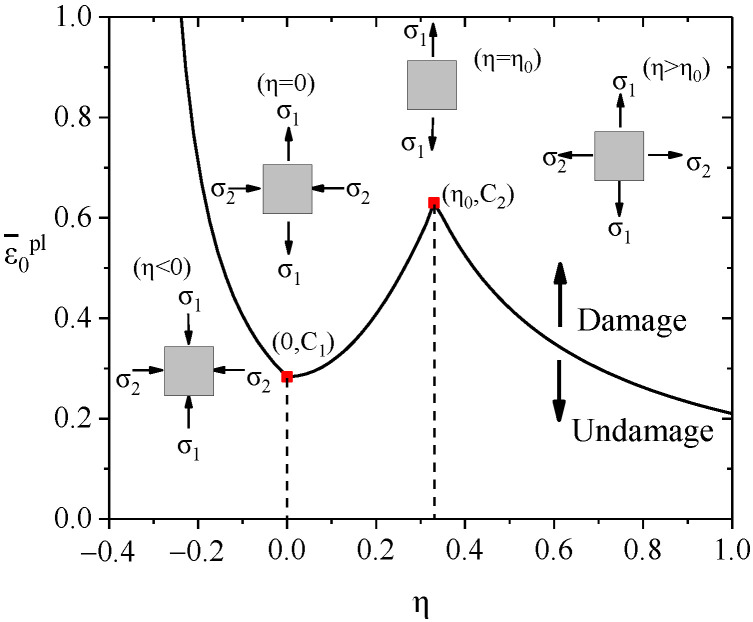
Equivalent damage plastic strain versus the degree of stress triaxial curves.

This paper adopts the simplified plastic damage model [32]. The equivalent plastic damage strain model of steel is defined in Equations Equations (5)–(7).

(5) $$\varepsilon_{0}^{\mathrm{pl}}=\left\{ \begin{array}{ll} \infty & \eta <\frac{1}{3}\\ C_{1}/(1+3\eta ) & -\frac{1}{3}\leq \eta <0\\ C_{1}+(C_{2}-C_{1}){\left(\eta /\eta_{0}\right)}^{2} & 0\leq \eta <\eta_{0}\\ C_{2}\eta_{0}/\eta & \eta_{0}<\eta \end{array}\right.$$

(6) $$C_{2}=-In(1-A_{R})$$

(7) $$C_{1}=C_{2}{\left(\frac{\sqrt{3}}{2}\right)}^{\frac{1}{n}}$$

where *C*_1_ is the equivalent damage plastic strain of steel under pure shear (*η* = 0); *C*_2_ is the equivalent damage plastic strain of steel under tension (*η* = *η*_0_), which can be defined by the necking area *A*_R_; n is the hardening coefficient of steel; and *n* = 0.226 in the FE model. *η*_0_ = 1/3 [33]. The simplified curve $\varepsilon_{0}^{\mathrm{pl}}-\eta$ curve is shown in Figure 9.

**Figure 10:**
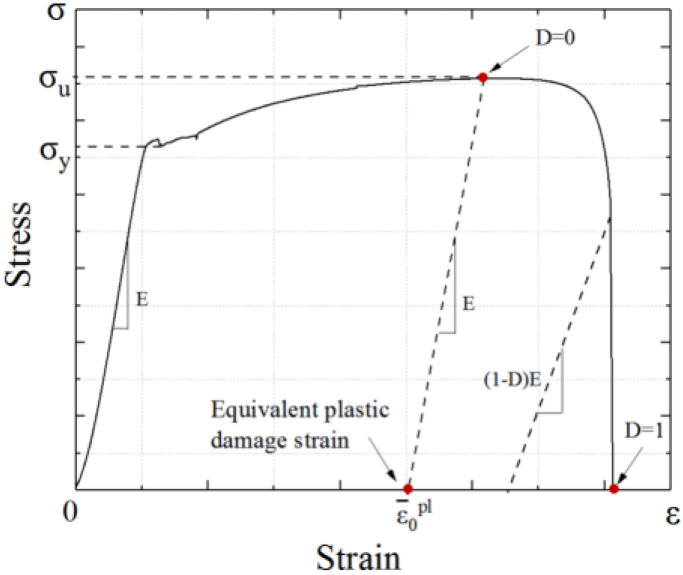
Determination of the equivalent plastic damage strain position.

The value of steel damage is related to the plastic displacement when the steel achieves the damage state, which is represented by the damage factor *D*_s_. The value of *D*_s_ ranges from 0 to 1. *D*_s_ = 0 indicates that no damage occurred to the steel, and the unloading stiffness don’t degrade. *D*_s_ = 1 indicates that the steel is completely destroyed, and the unloading stiffness drops to 0, as shown in Figure 10.

In this paper, the steel damage factor value *D* is adopted, as shown in Equation (8) [34]:

(8) $$D_{s}=1.3{\left(\frac{{\bar{u}}^{\mathrm{pl}}}{{\bar{u}}_{f}}\right)}^{7.6}$$

where ${\bar{u}}^{\mathrm{pl}}$ and ${\bar{u}}_{f}$ represent the plastic displacement and the ultimate displacement of the steel, respectively.

### 3.3. Concrete Constitutive Model

In the composite shear wall, the stress-strain curve of the infilled concrete is different from that of the plain concrete due to the constraint of the steel plate. The peak strain of the infilled concrete restrained by the steel tube is enhanced, and the descending stage is gentler because of the improved ductility [29]. The plastic damage model can be adopted to simulate the mechanical performance of concrete under cyclic loading in ABAQUS. The concrete constitutive model is calculated by the formulas presented in *Concrete Infilled by Steel Tube Structure-Theory and Practice* [35]. The formulas are shown in Equations (9) and (10).

(9) $$y=2x-x^{2}\;(x\leq 1)$$

(10) $$y=\left\{ \begin{array}{l} 1+q(x^{0.1\xi_{c}}-1)\;(\xi_{c}\geq 1.12)\\ \frac{x}{\beta {\left(x-1\right)}^{2}+x}\;(\xi_{c}<1.12) \end{array}\right.\;(x>1)$$

where $x=\frac{\varepsilon }{\varepsilon_{0}}$; $y=\frac{\sigma }{\sigma_{0}}$, $\xi =\frac{\alpha f_{y}}{f_{\mathrm{ck}}}$; $\alpha =\frac{A_{s}}{A_{c}}$; $q=\frac{\xi_{c}{}^{0.745}}{2+\xi_{c}}$; and $\beta =(2.36\times 10^{-5}){}^{\left[0.25+{\left(\xi_{c}-0.5\right)}^{7}\right]}f_{c}^{\prime 2}\times 3.51\times 10^{-4}$.

The stress–strain curve of concrete under tensile stress is defined by Equation (11).

(11) $$y=\left\{ \begin{array}{l} 1.2x-0.2x^{6}\;(x\leq 1)\\ \frac{x}{0.31\sigma_{p}{\left(x-1\right)}^{1.7}+x}\;(x>1) \end{array}\right.$$

where $x=\frac{\varepsilon }{\varepsilon_{p}}$; $y=\frac{\sigma }{\sigma_{p}}$, $\sigma_{p}=0.26{\left(1.25f_{c}{}^{\prime }\right)}^{2/3}$; and $\varepsilon_{p}=4.31\times 10^{-5}\sigma_{p}$.

*ξ*_c_ = Restrained coefficient factor.
*ε*_0_ = Peak strain of the concrete under uniaxial compression.
*σ*_p_ = Peak stress of the concrete under uniaxial tension.
$f_{c}^{\prime }$ = Cylinder compressive strength of the concrete.
*A*_s_ = Section area of the steel tube.
*A*_c_ = Section area of the infilled concrete.

The Poisson’s ratio of the concrete at the elastic stage is 0.2, and the elastic modulus is calculated by using Equation (12) [35]:

(12) $$E_{c}=4700\sqrt{f_{c}^{\prime }}$$

**Figure 11:**
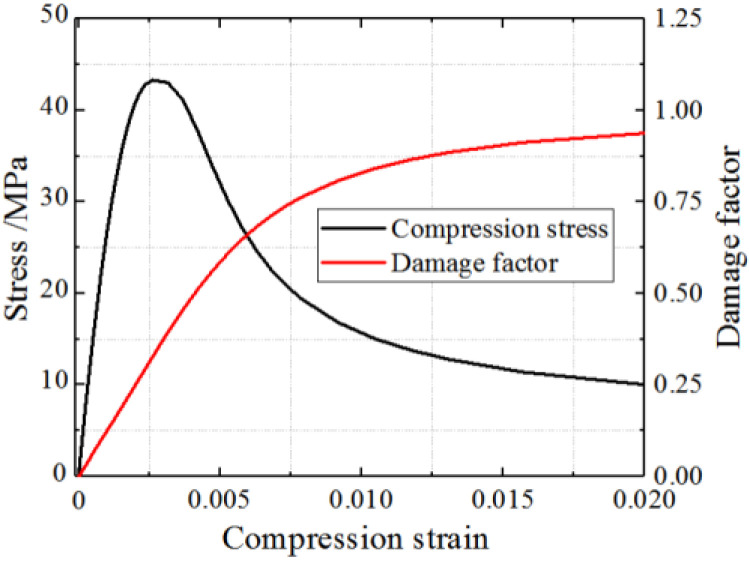
Concrete constitution model in compression.

**Figure 12:**
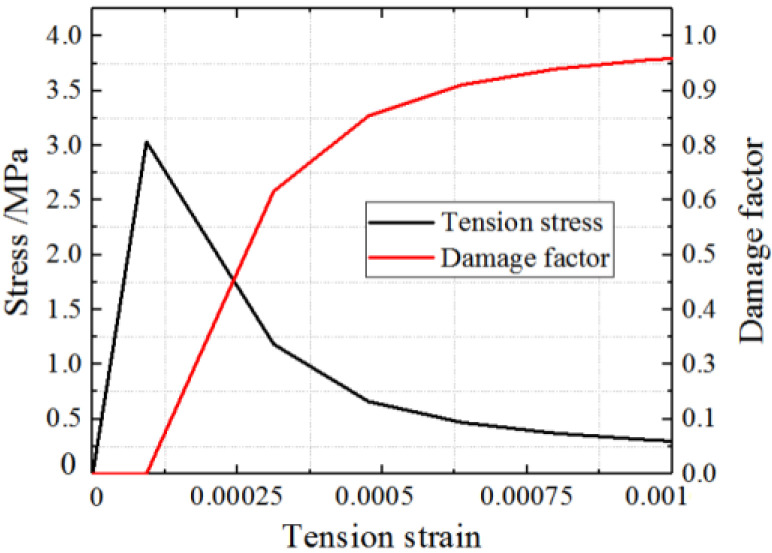
Concrete constitution model in tension.

Therefore, the constitutive curves of concrete under compression and tension are shown in Figure 11 and Figure 12.

The main parameters of the plastic damage model of concrete in ABAQUS are as follows: the expansion angle *Ψ* = 38°, the flow potential function eccentricity *ε* = 0.1, the ratio of the initial equivalent biaxial compressive strength to the initial uniaxial compressive strength *f*_b0_/*f*_c0_ = 1.16, the ratio of the second stress invariant of the meridional deflection under tension and compression *K*_c_ = 0.6, and the coefficient of viscosity *μ* = 0.001. 

**Figure 13:**
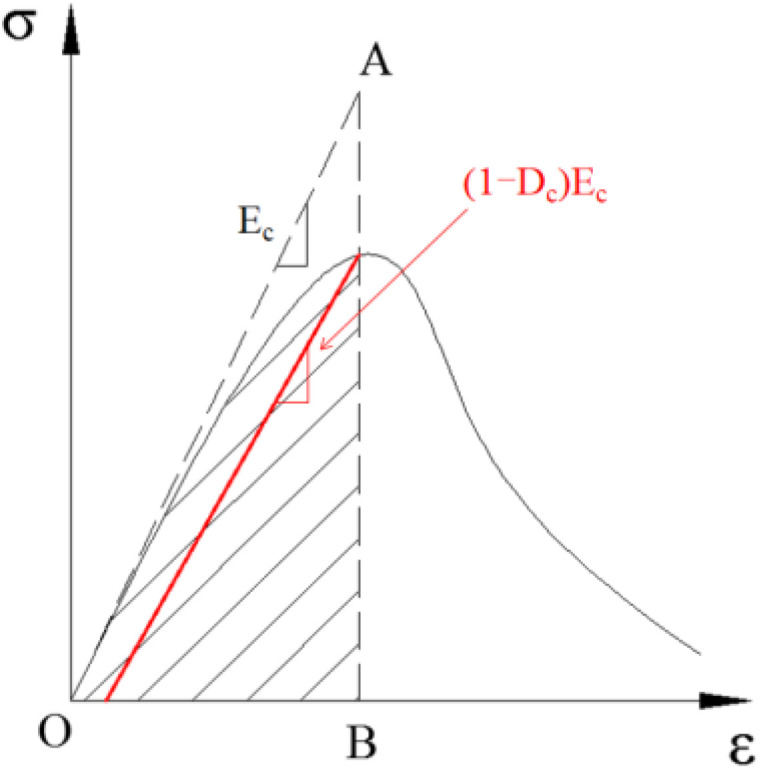
Damage model defined by the energy method.

The concrete cannot heal completely after cracks appeared, and the unloading stiffness changes. The damage factor *D*_c_ is used to describe the behaviour, as defined by Equation (13). As shown in Figure 13, the damage factor *D*_c_ is defined based on the energy method [36].

(13) $$D_{c}=1-\frac{S_{c}}{S_{0}}$$

*S*_c_ and *S*_0_ represent the shaded area and the area of triangle OAB, respectively. The damage factor of concrete is shown in Figure 11 and Figure 12. 

### 3.4. Meshing

**Figure 14:**
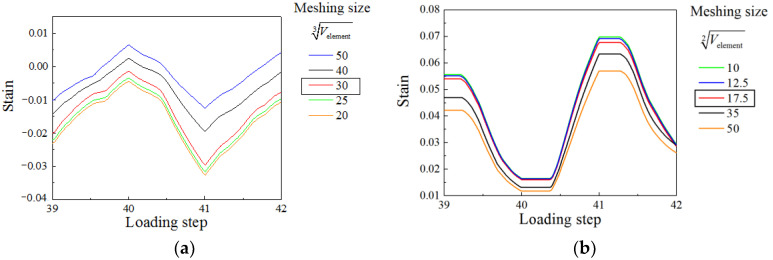
Strain history under different meshing sizes. (**a**) Concrete; (**b**) Steel plate.

Sensitivity analyses are carried out to determine the mesh size of steel plate and concrete. The equivalent lengths of solid element ($\sqrt[{3}]{V_{\mathrm{element}}}$) and shell element ($\sqrt[{2}]{V_{\mathrm{element}}}$) are used to describe the mesh size. Figure 14 shows the strain history of local buckling at the bottom of the wall under different equivalent lengths. The strain values remain constant when the equivalent length range from 20 to 30 and 10 to 17.5, respectively. Considering calculation efficiency and accuracy, $\sqrt[{3}]{V_{\mathrm{element}}}=30$ and $\sqrt[{2}]{V_{\mathrm{element}}}=17.5$ are selected as the mesh size for finite element models.

### 3.5. Simplification of the Finite Element Model

**Figure 15:**
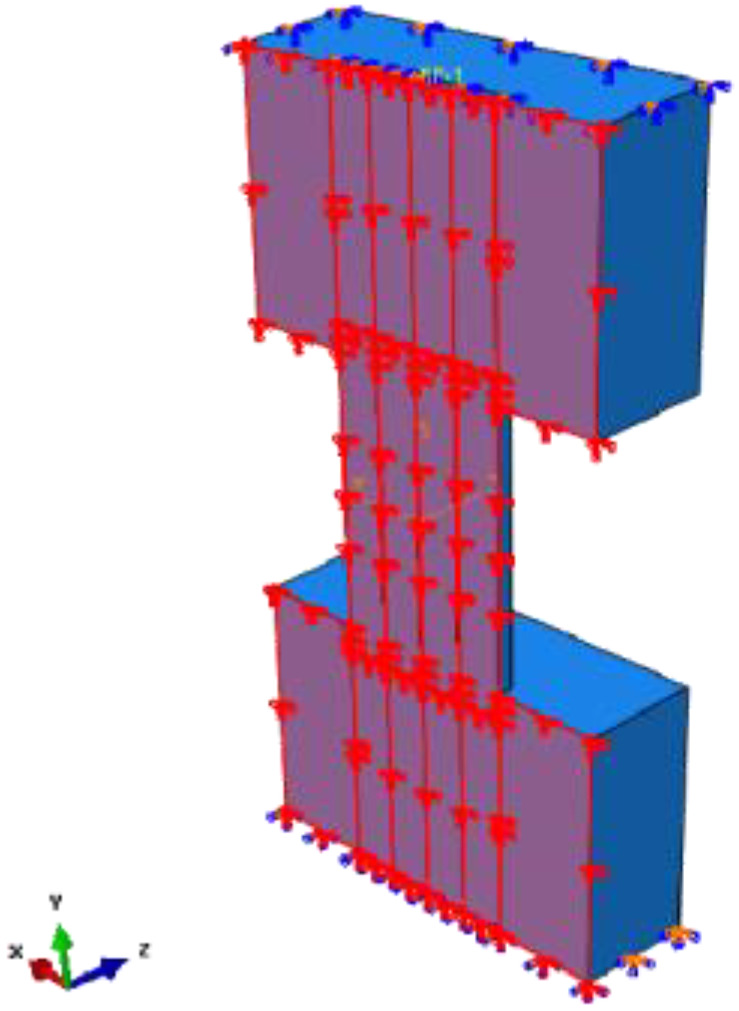
Constraint on the half model.

The FE model with more elements requires a longer calculation time. Thus, the whole model can be converted to a half model to ensure the efficiency of calculation. The displacement in the Z direction and the rotation in the X and Y directions are constrained on the half surface. The half model and surface constraints are shown in Figure 15. The lateral force of the half model is reduced by half in the same loading displacement compared with the full model. Therefore, the lateral force in the half model should be multiplied by 2 to compare with the test.

**Figure 16:**
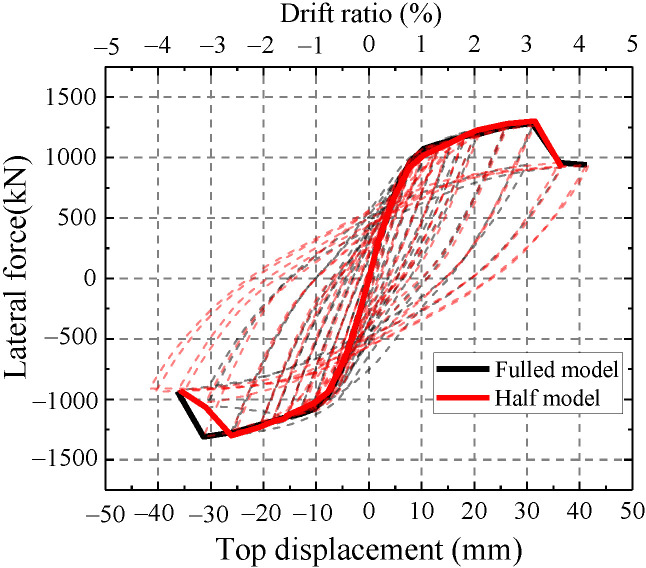
Comparison between the full model and half model.

As shown in Figure 16, the hysteretic curve and skeleton curve of the half model are similar to those of the overall model. Thus, the half model can simulate the hysteretic performance of the test. Half models are adopted to simulate the test in the following finite element analysis.

## 4. Validation of FE Models and Analysis of Results

### 4.1. Force-Displacement Curve

**Figure 17:**
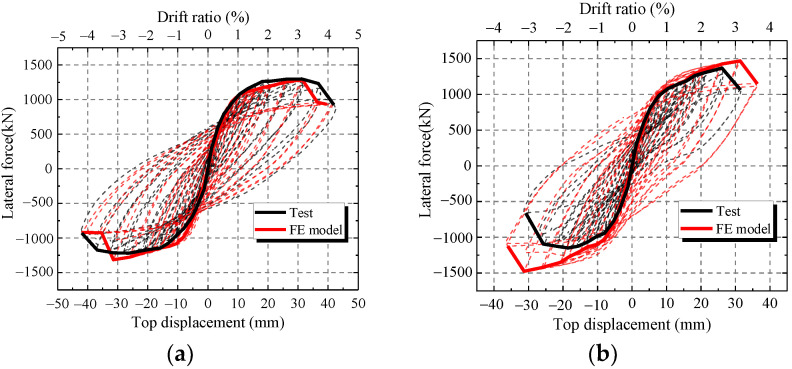

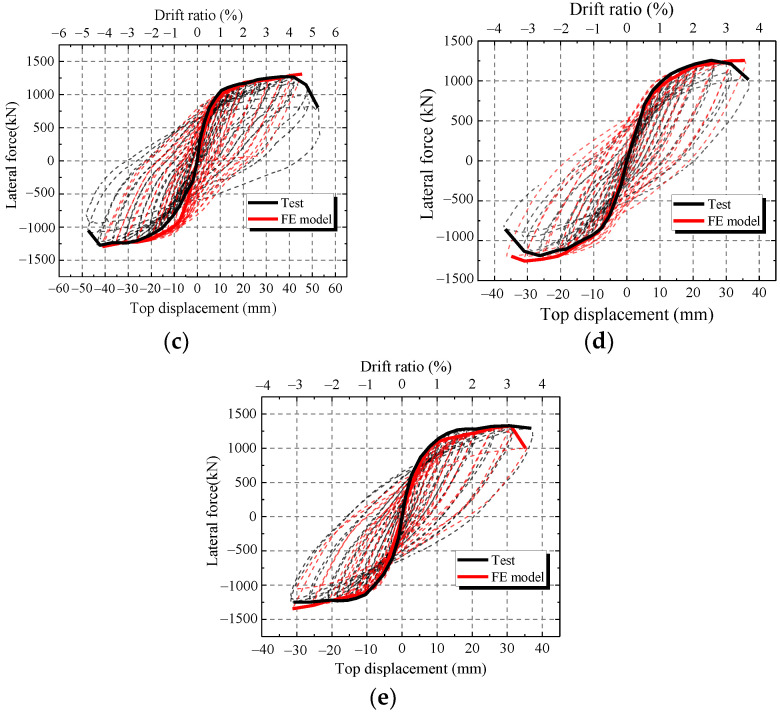
Comparison between the FE model and test. (**a**) CWSC-1; (**b**) CWSC-2; (**c**) CWSC-3; (**d**) CWSC-4; (**e**) CWSC-5.

To verify the accuracy of the FE model, the FE results of the hysteretic curves, skeleton curves and characteristic parameters are compared with those of the test. Figure 17 shows the comparisons of hysteretic curves and skeleton curves between the FE model and test. Figure 17 shows that the skeleton curves of FE model are similar to the test results at every loading level except CWSC-2. In addition, FE model accurately simulates the unloading path, especially at large horizontal displacements. Thus, the FE model can simulate the hysteretic curve of the composite shear wall.

As shown in Figure 17b, the ultimate strength capacity of CWSC-2 in the test is lower than that of the FE model. The weld fractured when the lateral force increased to a certain value in the test. The steel plate could not resist as a whole, and the lateral force decreased.

### 4.2. Characteristic Parameters

**Figure 18:**
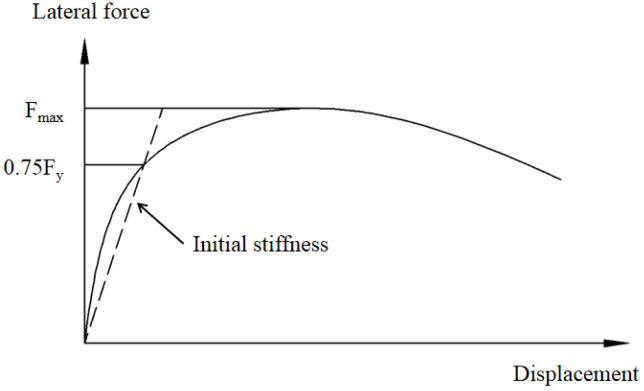
Initial stiffness definition method.

**Figure 19:**
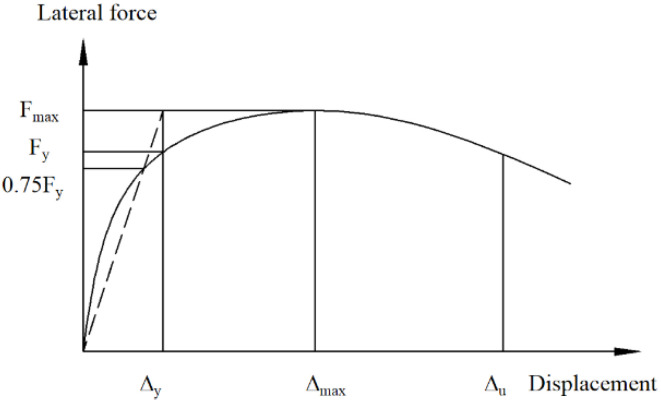
Yielding displacement definition method.

The characteristic parameters include the initial stiffness, ultimate strength, and ductility. The Li method [37] and the Park method [38] were adopted to calculate the initial stiffness and yielding displacement, respectively, as shown in Figure 18 and Figure 19. The ductility *μ* is defined as the ratio of the ultimate displacement to the yield displacement.

**Figure 20:**
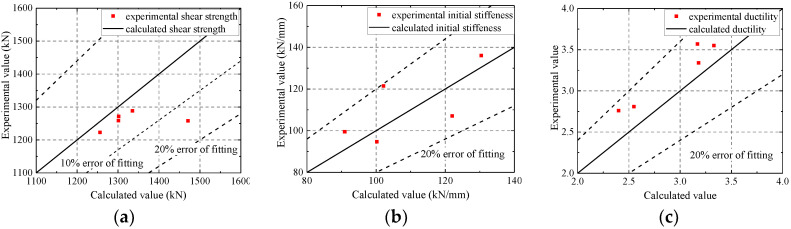
Errors of the characteristic parameters of the FE model and test. (**a**) Ultimate strength; (**b**) Initial stiffness; (**c**) Ductility.

As shown in Figure 20, FE model mostly overestimates the ultimate strength within an error of 10%. The error in the ultimate strength of CWSC-2 exceeds 10%, because the welds fractured when the lateral force reached the ultimate strength [12]. The fractures are the reason why the test samples have a lower seismic resistance. The FE model could simulate the initial stiffness and ductility of the composite shear wall well within an error of 20%. The FE model underestimates the ductility of the composite shear wall. Based on the analysis above, the FE model could well simulate the seismic behaviour of composite shear walls.

### 4.3. Failure Damage

In the test loading process, the web plate experienced severe local buckling at different positions with increasing horizontal displacement. The FE model could simulate the macro phenomenon. Since the failure damages of the samples were similar, the failure phenomenon of CWSC-1 was taken as an example for comparison with the simulation. 

**Figure 21:**
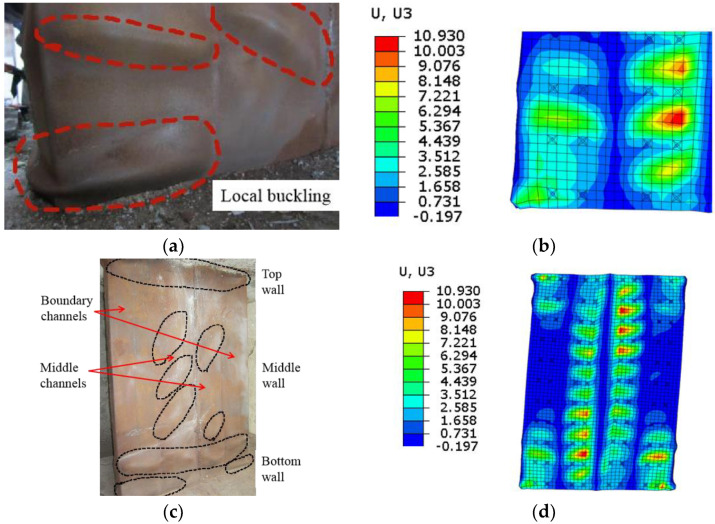
Comparison of the failure phenomena of CWSC-1 in the FE model and test. (**a**) Corner buckling in the test; (**b**) Corner buckling in the FE model; (**c**) Wall buckling in the test; (**d**) Wall buckling in the FE model.

Figure 21a,b show that when the ratio drift reaches 4.00%, severe local buckling occur in the bottom corner of the wall. The FE model simulates the local buckling in the corresponding position. Figure 21c shows that the overall buckling of the wall. The local buckling occur mostly on the top and bottom of the wall and the middle channels in middle wall. The bending moment and shear force in the boundary channels of the middle wall are small. The web plate experiences a small compression stress and do not undergo local buckling. The FE model could simulate the overall local buckling at the corresponding position. 

### 4.4. Mechanical Mechanism and Stress-Strain Analysis

#### 4.4.1. Distribution of the Lateral Force

**Figure 22:**
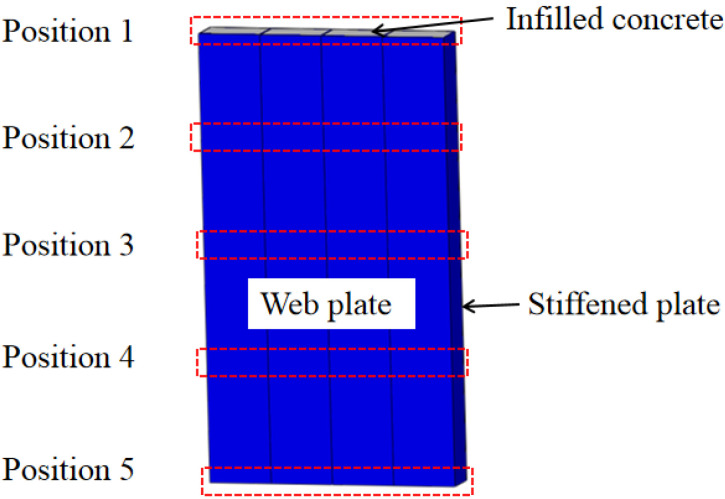
Position of the wall.

The lateral force is transferred from the top to the bottom along the height of wall. The lateral force of the wall is constant along the height of the wall. However, the bending moment is variable along the height of the wall, the distribution of lateral force in the web plate and concrete are different. To research the distribution of lateral force at the different horizontal displacements, five positions of the lateral force in the web plate and concrete are extracted, as shown in Figure 22. 

**Figure 23:**
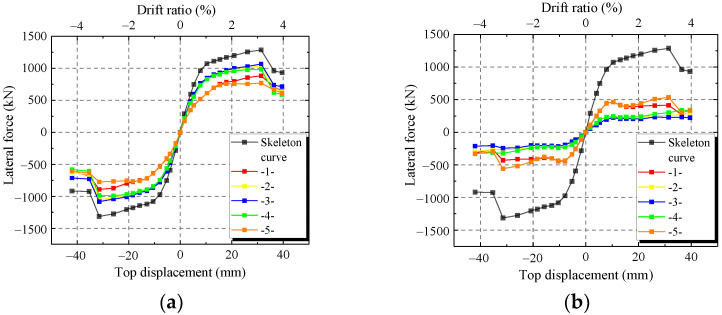
Lateral force in different positions of web plate and concrete. (**a**) Lateral force in web plate; (**b**) Lateral force in concrete.

Figure 23a,b show that the web plate and infilled concrete resist most of the lateral force. The lateral forces in the web plate and concrete are different at different positions. With increasing displacement to the bottom, the lateral force of web plate first increase and then decrease. The lateral force reaches a maximum value in the middle of web plate. In contrast, the lateral force of concrete shows a trend of decreasing first and then increasing. The lateral force reaches a minimum value in the middle of concrete. Because the top and bottom of wall generate the bending moment caused by the lateral force, the compositions of the top and bottom of wall are increased by the compression stress. The concrete resists more lateral force. With increasing displacement to the bottom, the bending moment and compression stress decrease. The lateral force in the concrete decreases but increases in the web plate.

**Figure 24:**
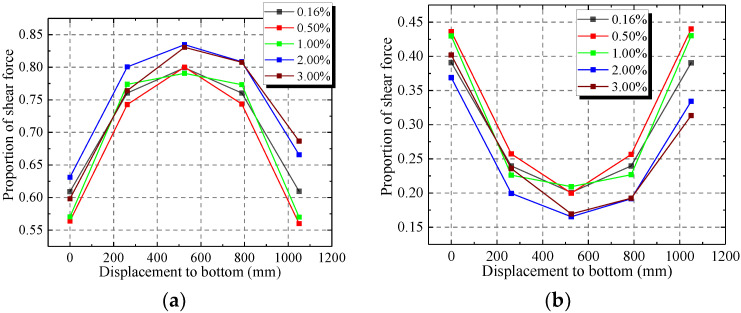
Proportion of lateral force in web plate and concrete. (**a**) Proportion in web plate; (**b**) Proportion in concrete.

Figure 24 shows that the middle of web plate has the largest proportion of lateral force, which ranges from 80% to 85%. The proportion of lateral force decreases from the middle of the web plate to the top and bottom. The proportion of lateral force in the top and bottom range from 55% to 70%. 

When the drift ratio increases from 0.16% to 0.50%, the proportion of lateral force of the web plate decreases but increases in the concrete. The stresses in the steel and concrete increase rapidly in the elastic-plastic stage. The stress increases faster in the concrete than the steel. Then, with the increase of drift ratio, the proportion of lateral force of the web plate increases, but the concrete decreases. The stress of concrete has reached ultimate stress. Then, the capacity of the bearing lateral force in concrete decreases. The steel reaches the hardening stage. Then, the stress of steel increases with increasing drift ratio. The added lateral force is resisted by the web plate. 

#### 4.4.2. Mechanical Mechanism of the Wall

**Figure 25:**
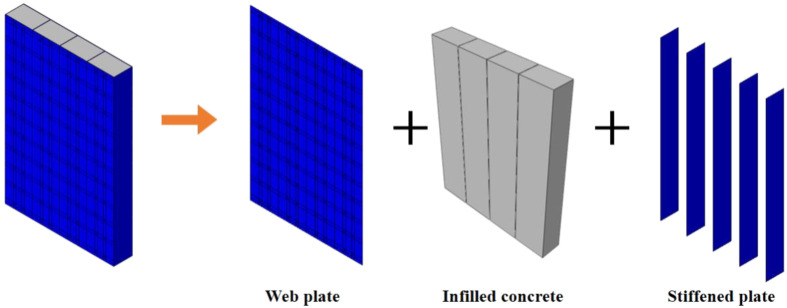
Component of the shear wall.

The shear wall is composed of three parts, including the web plate, infilled concrete, and stiffened plate, as shown in Figure 25. In Section 4.4.1, the web plate and infilled concrete resist most of the lateral force. The stiffened plate has a low contribution to resist the lateral force. Thus, only the mechanism of the web plate and infilled concrete could be analysed.

**Figure 26:**
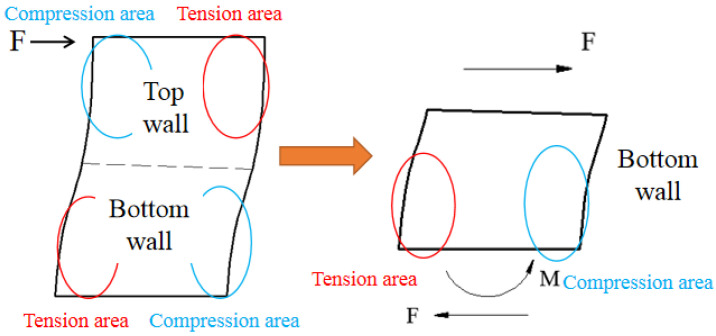
Simplified analysis of the shear wall.

The top and bottom concrete beams are almost not deformed in the loading process. Thus, the boundary conditions of wall include a sliding constant at the top and a fixed constant at the bottom. The stress state of the wall is antisymmetric under the lateral force. Thus, only the mechanism of the bottom wall could be analysed, as shown in Figure 26.

The mechanical behaviours of the shear wall are generally similar, so CWSC-1 is taken as an example for study.

**Figure 27:**
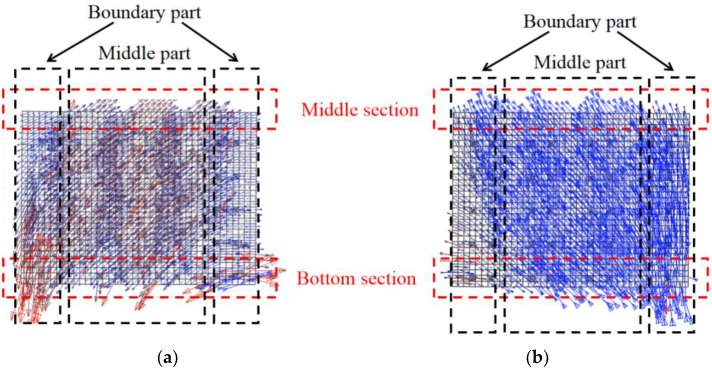
Principal stress vector diagram of web plate in yielding state. (**a**) Maximum principal stress vector diagram; (**b**) Minimum principal stress vector diagram.

This section studies the mechanical mechanism of the components under the cyclic load through the maximum and minimum principal stresses. Figure 27 describes the principal stress vector diagram of web plate in yielding state. The directions of the maximum and minimum principal stresses are almost vertical in the boundary parts of the bottom section. The directions of the maximum and minimum principal stresses have a certain angle with the horizontal stress in the middle part of the bottom section. The figures indicate that the bottom section mainly resists the bending moment and the middle section resists the shear force. The direction of the maximum and minimum principal stresses have a certain angle with the horizontal stress in the middle section, which indicates that the middle section resists the shear force.

**Figure 28:**
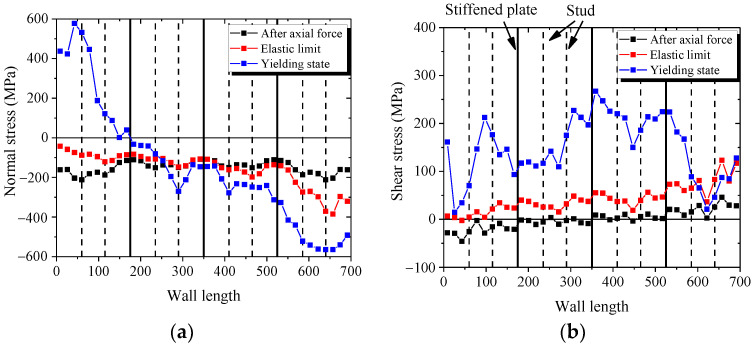

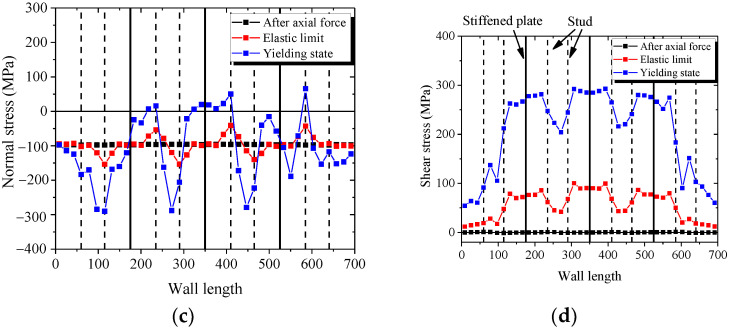
Normal stress and shear stress of the wall. (**a**) Normal stress in the bottom section; (**b**) Shear stress in the bottom section; (**c**) Normal stress in the top section; (**d**) Shear stress in the top section.

Figure 28a,b presents the distribution of normal stress and shear stress in the bottom section of wall. Figure 28a shows that the bottom section almost uniformly borne the compressive stress after axial force. With increasing lateral force, the normal stresses in the boundary parts of bottom section increase, but the middle part of bottom section is almost constant. Figure 28b shows that the shear stresses in the boundary parts of bottom section are almost equal to zero after the axial force is applied. With increasing lateral force, the normal stresses in the boundary parts of bottom section are almost constant, and the shear stress in the middle part of bottom section increases. The figures indicate that the mechanical mechanism in the bottom section of the wall is borne of the bending moment, shear force, and axial force. The bending moment is resisted by the boundary parts, and the shear force is resisted by the middle part.

Figure 28c,d describe the distribution of normal stress and shear stress in the middle section of wall. Figure 28c shows that the middle section uniformly resist the compressive stress after the axial force is applied. With increasing lateral force, the normal stress in the middle section is almost constant. Figure 28d shows that with increasing lateral force, the shear stresses in the middle part of middle section increase rapidly, and the shear stresses in the boundary parts of middle section increase almost slowly. These figures indicate that the bending moment in the middle section of wall is equal to zero. The mechanical mechanism in middle section and middle part is resisting the shear force and the boundary part is resisting the axial force. 

**Figure 29:**
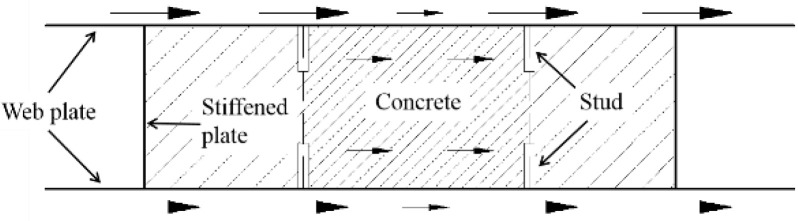
Transferred path of the shear force.

The shear stresses of the web plate mainly distribute around the stiffened plate and decrease at the stud. Because the studs connect the concrete and the web plate, part of the shear force transfer to the internal concrete through studs. Therefore, when the wall is subjected to lateral force, the shear force of the web plate between the studs is less than the stiffened plate. Another important reason is that the stiffened plate could prevent the web plate from buckling, so the web plate near the stiffened plate could carry more shear force, as shown in Figure 29.

**Figure 30:**
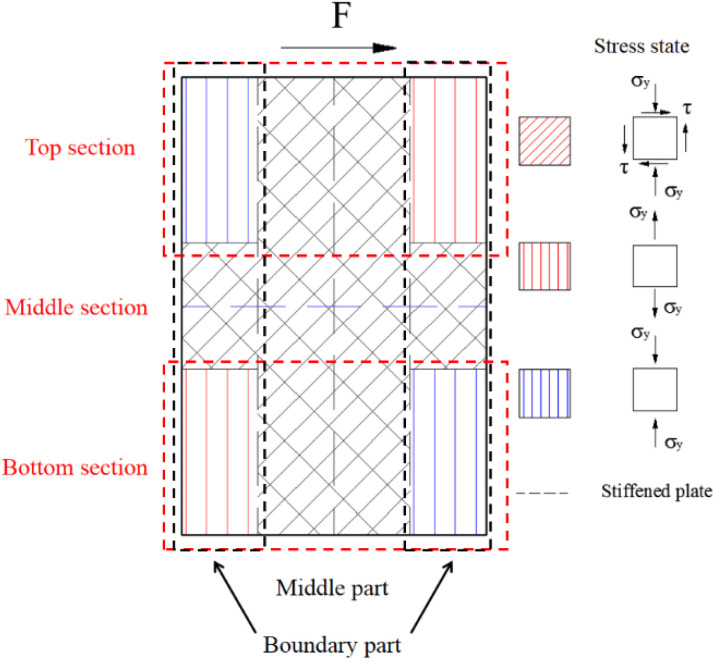
Stress state of the web plate.

Figure 30 shows the stress state of the web plate proposed by analysing the mechanical mechanism of web plate. The middle part and boundary parts of the middle section bear the shear force to transmit the lateral force, and the boundary parts of the top and bottom sections bear the tension and compression force to resist the bending moment by the lateral force. 

**Figure 31:**
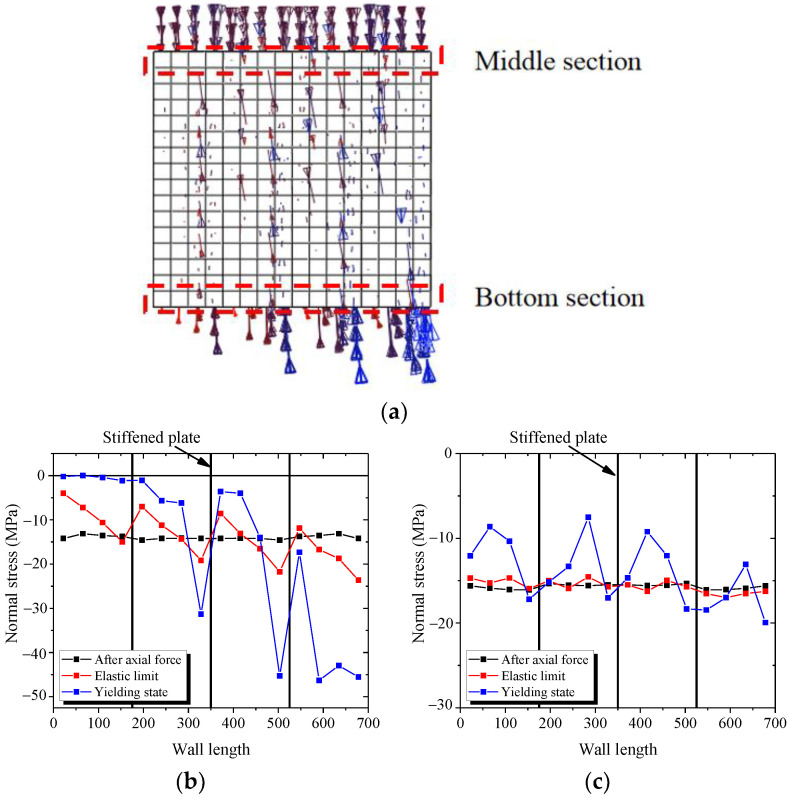
Normal stress in the concrete. (**a**) Minimum principal stress vector diagram; (**b**) Normal stress of the bottom section; (**c**) Normal stress of the middle section.

As shown in Figure 31a, the minimum principal stress of concrete is almost vertical, which indicates that the concrete mainly resists the vertical force.

As shown in Figure 31b,c, the normal stress of the middle section is almost unchanged with increasing drift ratio. Because the middle of wall mainly bears the shear force and axial force, the shear force of concrete is small. The vertical stress is almost constant. 

The normal stress at the bottom section is divided into four sections, each of which increases from left to right. The concrete wall is separated into several concrete columns by the stiffened plates, each of which is independent and resists the lateral force alone. Thus, the vertical stress in the bottom section of each concrete column develops gradually from the left to right under the lateral force and axial force. 

## 5. Parametric Analysis

To simulate the parameters of shear walls in actual high-rise buildings, parametric analysis is conducted based on the section size and design parameters of the shear walls in a proposed high-rise building [39]. The influence rules of the different parameters are studied, and formulas for the bearing capacity and stiffness of the composite shear wall are proposed. In order to study the hysteric behavior of model in parametric analysis, the cyclic load is applied to the wall. The model boundary condition is consistent with the test. 

The parameters include the wall thickness, steel ratio, shear span ratio, axial compression ratio, concrete strength, steel strength and channel length-to-width ratio. A standard model is used as a reference, and then the parameters are changed to conduct parametric analysis. The standard model parameters are chosen as follows: the wall thickness is 1200 mm, the steel ratio is 5%, the shear span ratio is 0.5, the axial compression ratio is 0.5, the axial compressive strength of the concrete is 60 MPa, the yield strength of the steel is 345 MPa, and the length-to-width ratio of the channel is 2.

In the parametric analysis, the wall thickness ranges from 600 mm to 1500 mm, the steel ratio ranges from 3.3% to 8.3%, the shear span ratio ranges from 0.3 to 1.5, the axial compression ratio ranges from 0.2 to 0.6, the axial compressive strength of the concrete ranges from 40 MPa to 100 MPa, the yield strength of the steel ranges from 235 MPa to 420 MPa, and the length-to-width ratio of the channel ranges from 1 to 3.

### 5.1. Influence Rules of the Parameters

The hysteretic curve and skeleton curve are obtained by each model. The influence rules of key design parameters are studied by skeleton curve, including elastic stiffness, ultimate strength capacity, and ductility.

#### 5.1.1. Influence on the Wall Thickness

**Figure 32:**
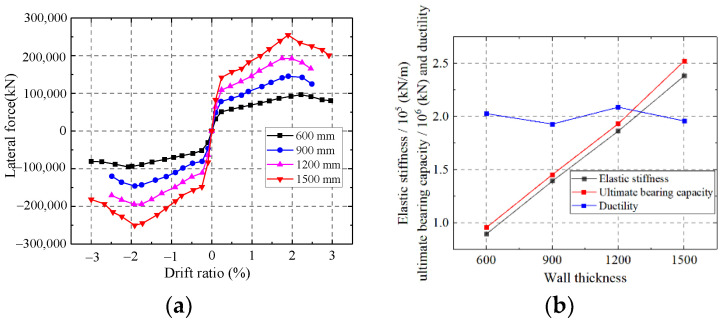
Influence on the wall thickness. (**a**) Skeleton curves of the model; (**b**) Comparison of key parameters.

As shown in Figure 32, the elastic stiffness and ultimate strength capacity of the wall enhance with increasing wall thickness, but improving the wall thickness has little effect on the ductility. Increasing the wall thickness could improve the section area of the wall. However, increasing the wall thickness only increases the thickness of the infilled concrete, which contributes slightly to the ductility of the composite shear wall.

#### 5.1.2. Influence on the Steel Ratio

**Figure 33:**
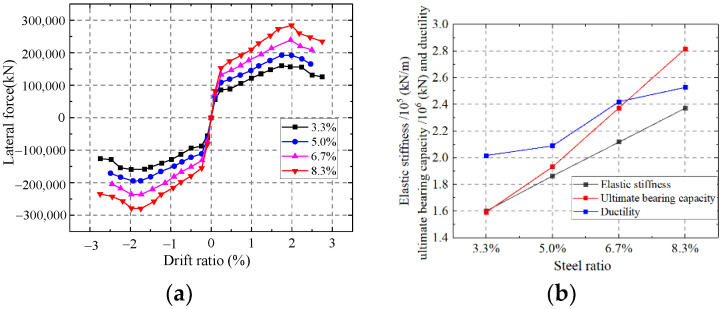
Influence on the steel ratio. (**a**) Skeleton curves of the model; (**b**) Comparison of key parameters.

As shown in Figure 33, the elastic stiffness, ultimate strength capacity and ductility of the wall are enhanced with the increase of the steel ratio. Increasing the steel ratio could improve the flexural rigidity of the wall and the section area of the steel. The steel plate in the wall could resist most of the lateral force. The ultimate strength capacity and ductility are improved significantly with the increasing steel ratio.

#### 5.1.3. Influence on the Shear Span Ratio

**Figure 34:**
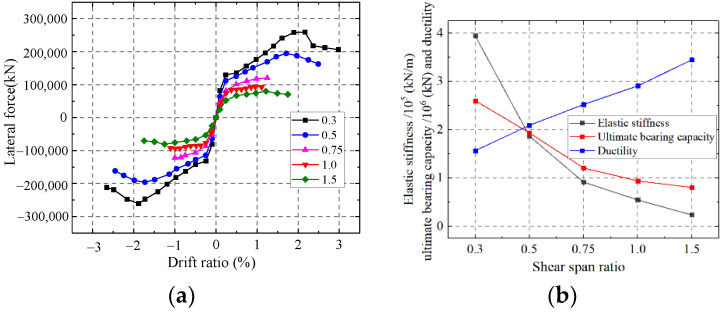
Influence on the shear span ratio. (**a**) Skeleton curves of the model; (**b**) Comparison of key parameters.

As shown in Figure 34, the elastic stiffness and ultimate strength capacity of the wall weaken but the ductility enhances with an increase in the shear span ratio. Increasing the shear span ratio changes the mechanical mode of the composite shear wall. In addition, the failure mode changes from relatively rigid shear failure to relatively flexible flexural failure. The elastic stiffness and bearing capacity enhance but the ductility weakens.

**Figure 35:**
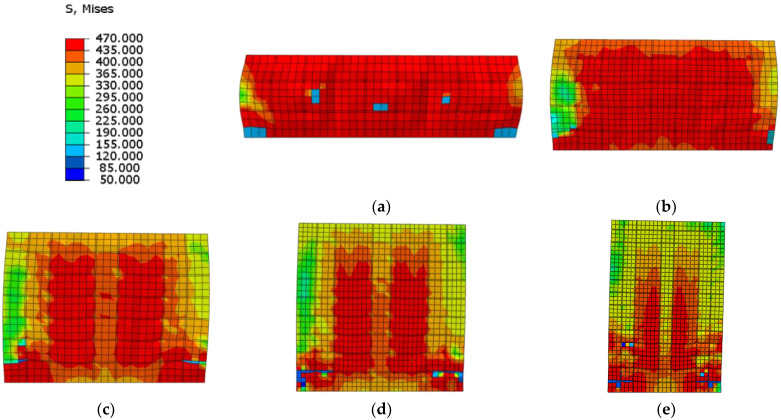
Stress distribution of web plate at the ultimate strength. (**a**) λ = 0.3; (**b**) λ = 0.5; (**c**) λ = 0.75; (**d**) λ = 1.0; (**e**) λ = 1.5.

Compared to the stress distribution in different shear span ratios at the ultimate strength capacity (Figure 35), the Mises stress of web plate almost reaches ultimate strength in lower shear span ratio, and the web plate approximately reaches plane shear state. With the increase of shear span ratio, parts of the web plate cannot reach the ultimate strength, or even exceed the yield strength. The results indicate the web plate cannot completely develop plasticity when the higher shear span ratio wall reaches the ultimate strength capacity. Because the higher shear span ratio wall is controlled by the bending-shear failure mode, and the stress is small at the top of wall where the bending moment is small. Therefore, the shear span ratio of composite shear wall needs to be no more than 0.75.

#### 5.1.4. Influence on the Axial Compression Ratio

**Figure 36:**
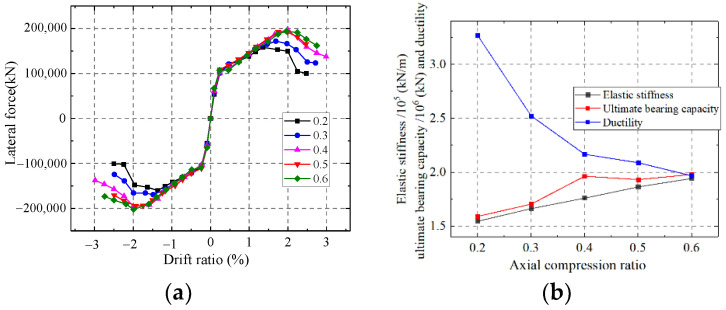
Influence on the axial compression ratio. (**a**) Skeleton curves of the model; (**b**) Comparison of key parameters.

As shown in Figure 36, the elastic stiffness and ultimate strength capacity of the wall enhance but the ductility weakens with an increase in the axial compression ratio. A larger axial compression ratio could increase the interaction between the steel and concrete, which improves the elastic stiffness and ultimate strength. However, the larger compression force tends to cause the shear wall damage rapidly after the ultimate strength and ductility weaken. The ductility weakens with an increase in the axial compression ratio.

#### 5.1.5. Influence on the Axial Compressive Strength of Concrete

**Figure 37:**
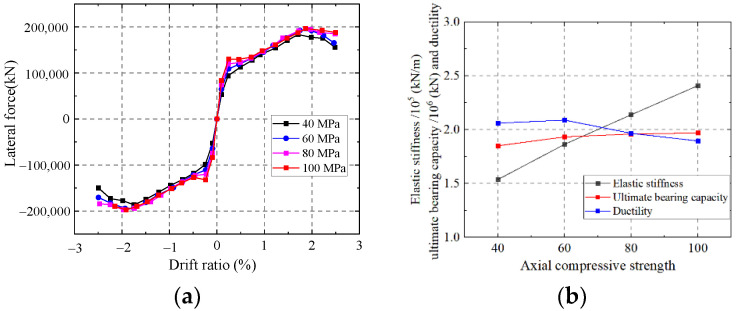
Influence on the axial compressive strength of concrete. (**a**) Skeleton curves of the model; (**b**) Comparison of key parameters.

As shown in Figure 37, the elastic stiffness of the wall enhances with an increase in the axial compressive strength of the concrete. Increasing the axial compressive strength of concrete has little effect on the ductility and ultimate strength of the wall, and the elastic modulus improves. While increasing the ultimate strength capacity and ductility are relevant to increase the steel ratio. Thus, increasing the axial compressive strength of concrete has little effect on the elastic stiffness and ductility.

#### 5.1.6. Influence on the Yield Strength of Steel

**Figure 38:**
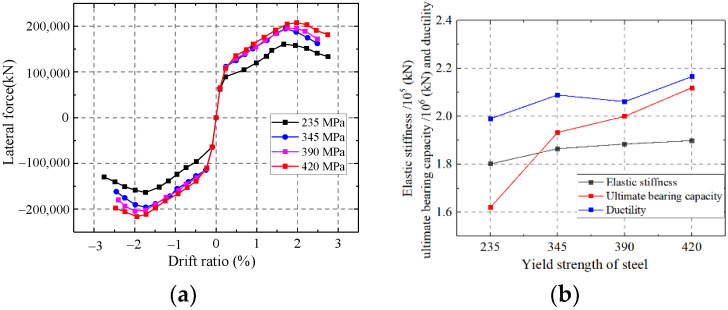
Influence on the yield strength of steel. (**a**) Skeleton curves of the model; (**b**) Comparison of key parameters.

As shown in Figure 38, the ultimate strength capacity of the wall enhances with increasing yield strength of steel. Increasing the yield strength of steel has little effect on the ductility and elastic stiffness. Increasing the yield strength of steel could not improve the elastic modulus and ductility of steel. Therefore, increasing the yield strength of steel could not improve the elastic stiffness and ductility of the wall.

#### 5.1.7. Influence on the Length-to-Width Ratio of the Channel

**Figure 39:**
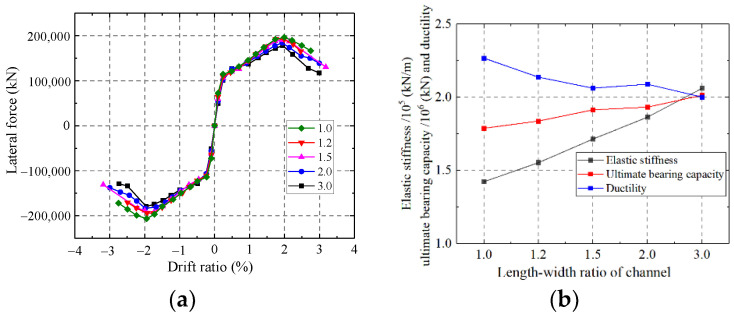
Influence on the length-width ratio of the channel. (**a**) Skeleton curves of the model; (**b**) Comparison of key parameters.

As shown in Figure 39, the elastic stiffness and ultimate strength capacity enhanced, but the ductility weakened with the increase in the length-to-width ratio of the channel. With increasing the channel length-to-width ratio, the mechanical mode of infilled concrete changed from multi-section columns to a whole wall. Therefore, increasing the length-to-width ratio of the channel could enhance the elastic stiffness and ultimate strength capacity of the wall but weaken the ductility.

**Figure 40:**
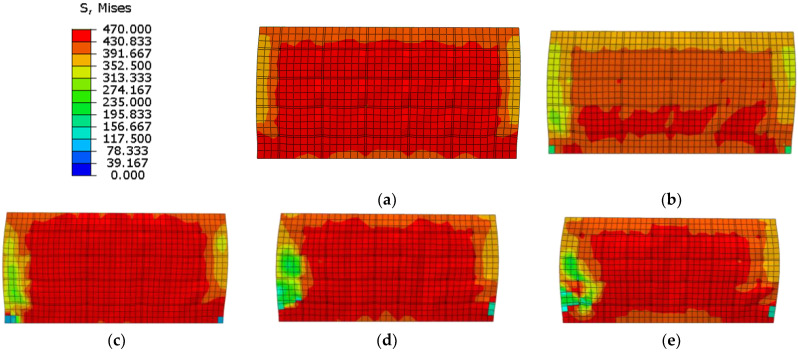
Stress distribution of web plate at the ultimate strength. (**a**) ξ = 1.0; (**b**) ξ = 1.2; (**c**) ξ = 1.5; (**d**) ξ = 2.0; (**e**) ξ = 3.0.

Compared the stress distribution in different length-to-width ratio of the channel at the ultimate strength (Figure 40), the Mises stress is large in the middle part and small in the boundary part. The results indicate the wall is controlled by the bending-shear failure mode. With the decrease of length-to-width ratio of the channel, the area of shear stress area is larger, while the tension and compression stress areas are smaller due to the separation by stiffened plates. The stress distribution of web plate is more uniform when the length-to-width ratio of the channel decreases, and the performance of wall could be fully developed. Therefore, the length-to-width ratio of the channel in the composite shear wall needs to be no more than 1.2.

### 5.2. Formulation for Bearing Capacity and Stiffness Prediction

The composite shear wall is usually designed by considering two aspects: (1) the elastic stiffness *K*_0_ should be considered in elastic design, and (2) the yielding bearing capacity *F*_y_, yielding section stiffness *K*_y_, and ultimate strength capacity *F*_max_ are studied in elastic-plastic design. 

#### 5.2.1. Prediction of the Ultimate Strength

The following are assumed to identify the resistance of the composite shear wall: (1) The section of the wall remains planar after flexural bending. (2) The tension of the concrete on the tension side is ignored. The compressive strength of the concrete on the compression side is reached, and the yield strength of the steel plate is reached. (3) The restrained action of the steel plate on the concrete is considered. (4) The steel plate and concrete fully interact.

**Figure 41:**
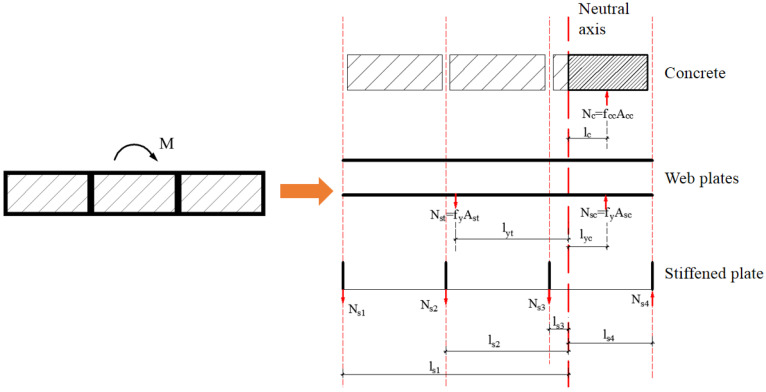
Section stress distribution of the composite shear wall.

Based on the above assumptions, the section stress distribution of the shear wall under horizontal and axial compression forces is shown in Figure 41.

According to the resultant force equilibrium equation, Equations (14) and (15) could be determined.

(14) $$f_{\mathrm{cc}}\cdot A_{\mathrm{cc}}+f_{y}\cdot A_{\mathrm{sc}}+N_{\mathrm{si}}=f_{y}\cdot A_{\mathrm{st}}+\sum_{i=1}^{4}\left(N_{\mathrm{si}}\cdot l_{\mathrm{si}}\right)$$

(15) $$M_{u}=f_{\mathrm{cc}}\cdot A_{\mathrm{cc}}\cdot l_{c}+f_{y}\cdot A_{\mathrm{sc}}\cdot l_{\mathrm{yc}}+f_{y}\cdot A_{\mathrm{st}}\cdot l_{\mathrm{yt}}+\sum_{i=1}^{4}\left(N_{\mathrm{si}}\cdot l_{\mathrm{si}}\right)$$

$$f_{\mathrm{cc}}=f_{c}\cdot \left(1+1.8\xi_{\mathrm{sc}}\right)$$

$$\xi_{\mathrm{sc}}=\frac{f_{y}\cdot A_{s1}}{f_{c}\cdot A_{c1}}$$

*f*_cc_ = Axial compressive strength of the constrained concrete [40].
*f*_c_ = Axial compressive strength of concrete.
*f*_y_ = Yield strength of steel.
*ξ*_sc_ = Constraint coefficient of the concrete in the CWSC sample.
*A*_s1_ = Area of the constrained steel plate.
*A*_c1_ = Area of the infilled concrete.
*A*_cc_ = Area of the concrete under uniaxial compression.
*A*_sc_ = Area of the steel plate under uniaxial compression.
*A*_st_ = Area of the tension steel plate.
*N*_si_ = Vertical force on the stiffened plate.
*l*_si_ = Distance from the stiffened plate to the neutral axis.
*l*_c_ = Distance from the constrained concrete to the neutral axis.
*l*_yc_ = Distance from the compression steel plate to the neutral axis.
*l*_yt_ = Distance from the tension steel plate to the neutral axis.

As the composite shear wall is subject to the coupling action of axial force, shear force and bending moment, the interaction of three factors needs to be considered when calculating the ultimate strength [41]. The formula used to calculate the ultimate strength capacity of the composite shear wall is proposed in Equation (16).

(16) $$-\frac{N}{N_{u}\cdot 2.17}+{\left[\frac{M}{M_{u}\left(3.03\xi +0.3\right)}\right]}^{2}+{\left(\frac{V}{V_{u}}\right)}^{2}=1$$

(17) $$N_{u}=f_{c}\cdot A_{c}+f_{y}\cdot A_{s}$$

(18) $$V_{u}=1.99\cdot \sqrt{f_{c}^{\prime }}\cdot A_{c}+\frac{f_{y}}{\sqrt{3}}\cdot A_{\mathrm{sw}}$$

*N* = Axial force applied at the shear wall.
*M* = Bending moment applied at the shear wall.
*V* = Horizontal shear force applied at the shear wall.
*N*_u_ = Axial bearing capacity of the shear wall, determined by Equation (17).
*M*_u_ = Flexural bearing capacity of the shear wall, determined by Equation (15).
*V*_u_ = Ultimate strength capacity of the shear wall, determined by Equation (18) [42].
*ξ* = Ratio of the channel width to wall width.
$f_{c}^{\prime }$ = Compressive strength of the cylinder concrete.
*A*_sw_ = Steel plate area parallel to the horizontal force.

**Figure 42:**
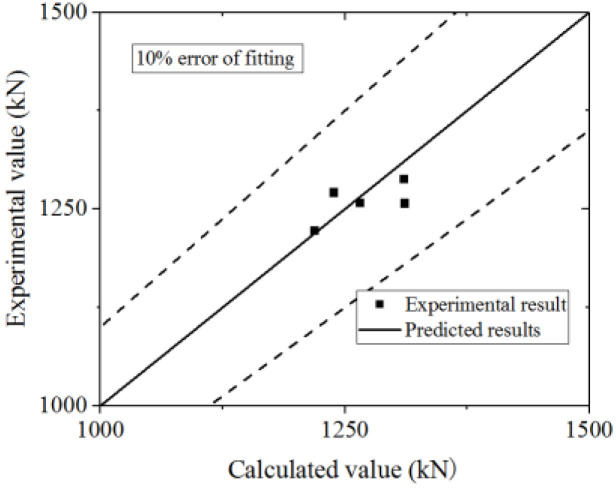
Comparison of ultimate strength capacity in the formula and test.

**Figure 43:**
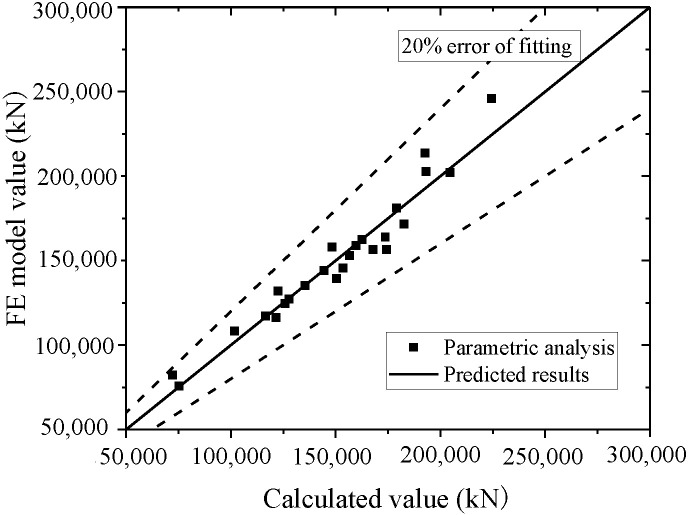
Comparison of ultimate strength capacity in the formula and parametric analysis.

Equation (16) is used to fit the test and FE model results. The test and parametric analysis results are compared to the formula results, as shown in Figure 42 and Figure 43. The formula could fit the test with an error less than 10%, and fit the parametric analysis with an error less than 20%. Thus, the formula could accurately predict the ultimate strength capacity.

**Figure 44:**
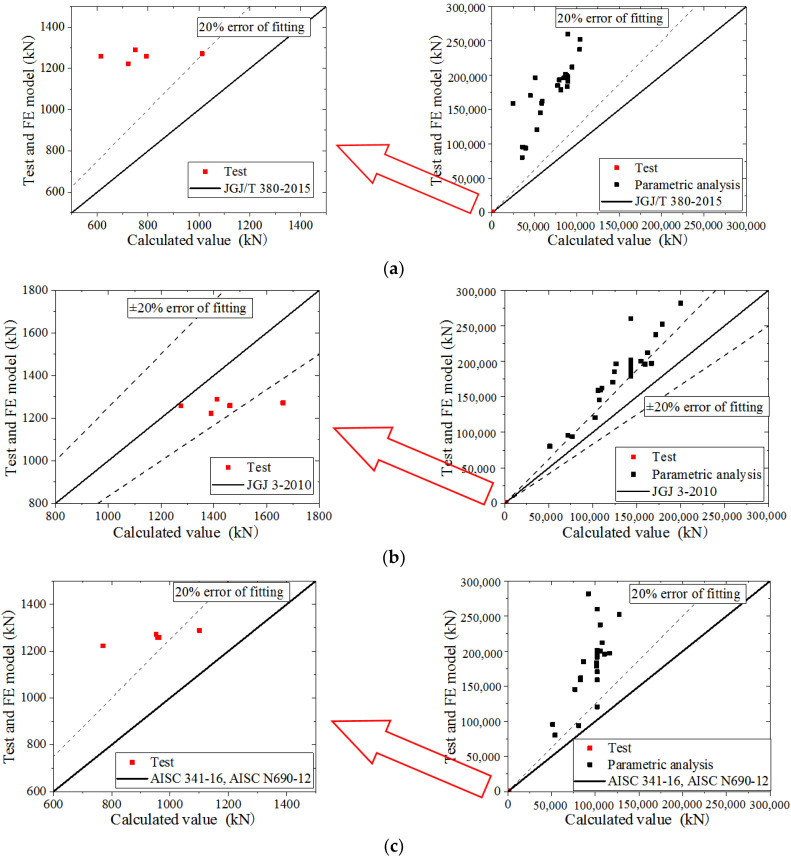
The comparison with the specifications. (**a**) JGJ/T 380-2015; (**b**) JGJ 3-2010; (**c**) AISC 341-16 and AISC N690-12.

The specification formulas used to calculate the ultimate bearing capacity, including the JGJ/T 380-2015 [27], JGJ 3-2010 [43], AISC 341-16 [44], and AISC N690-12 [25], compared with the test and parametric analysis, as shown in Figure 44a,c, show that the values in the test and parametric analysis are higher than the values obtained by the formulas and exceed 20% error, indicating that the ultimate bearing capacity is seriously underestimated by the JGJ/T 380-2015, AISC 341-16, and AISC N690-12. Figure 44b shows that the values in the test are lower than values obtained by the formula, within the 20% error. Most values obtained in the parametric analysis are higher than the those obtained with the formulas and exceed 20% error, indicating that the ultimate bearing capacity obtained in the parametric analysis is underestimated by JGJ 3-2010. Therefore, the formula in this paper could predict the ultimate bearing capacity of CWSC more accurately. 

#### 5.2.2. Prediction of the Yielding Bearing Capacity

The same method is used to calculate the yielding bearing capacity of the composite shear wall. The formula used to calculate the yielding bearing capacity of the composite shear wall is proposed in Equation (19).

(19) $$-\frac{N}{N_{u}\cdot 0.6}+{\left[\frac{M}{M_{u}\left(0.4\xi +0.55\right)}\right]}^{2}+{\left(\frac{V}{V_{u}}\right)}^{2}=1$$

The symbols in Equation (19) are defined in Section 5.2.1.

**Figure 45:**
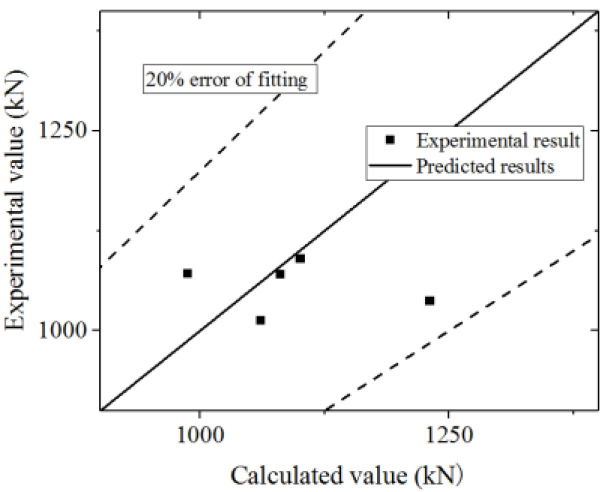
Comparison of the yielding bearing capacities in the formula and test.

**Figure 46:**
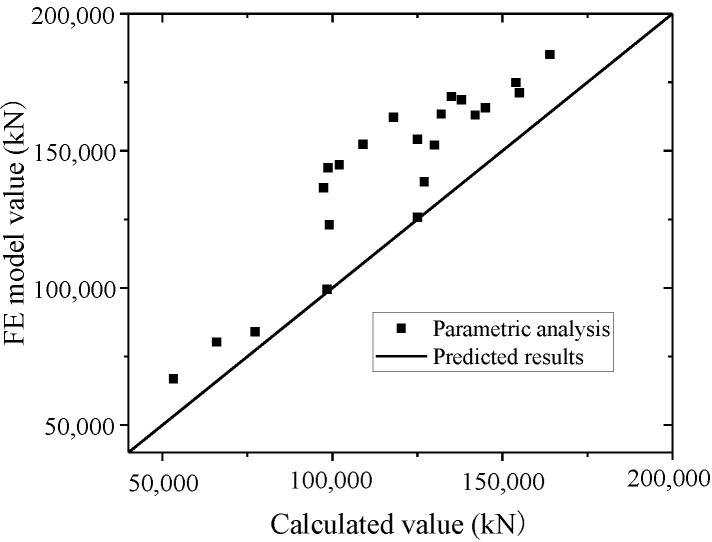
Comparison of the yielding bearing capacities in the formula and parametric analysis.

Equation (19) is used to fit the test and FE model results. The test and parametric analysis results are compared with the formula results, as shown in Figure 45 and Figure 46. The formula could fit the test with an error less than 20%. The values of the FE model are higher than those of the formula, which indicates that the formula could conservatively predict the FE model results. Thus, the formula could accurately predict the yielding bearing capacity.

#### 5.2.3. Prediction of the Elastic Stiffness

According to previous research [12], the elastic stiffness *K*_0_ of the composite shear wall is composed of the flexural stiffness *K*_m_ and shear stiffness *K*_v_. The formula for elastic stiffness is shown in Equations (20)–(22).

(20) $$\frac{1}{K_{0}}=\frac{1}{K_{m}}+\frac{1}{K_{v}}$$

(21) $$K_{m}=\frac{p\cdot E_{\mathrm{sc}}I_{\mathrm{sc}}}{h^{3}}$$

(22) $$K_{v}=\frac{G_{\mathrm{sc}}A_{\mathrm{sc}}}{k\cdot h}$$

*p* = End constraint coefficient of the sample, where *p* = 3 in the FE model.
*k* = Section influence coefficient, where *k* = 1.2.
*h* = Height of the wall.
*E*_sc_*I*_sc_ = Flexural stiffness of the shear wall, where *E*_sc_*I*_sc_ = *E*_s_*I*_s_ + *E*_c_*I*_c_.
*G*_sc_*A*_sc_ = Shear stiffness of the shear wall, where *G*_sc_*A*_sc_ = *q*_e_(*G*_s_*A*_s_ + *G*_c_*A*_c_).
*q*_e_ represents the shear stiffness reduction coefficient, as proposed by Equation (23).

(23) $$q_{e}=0.09-2.04\cdot {\left(b/B\right)}^{2}+2.10\cdot (b/B)-0.40n^{2}+0.65n+0.16\lambda^{2}-0.6\lambda$$

where *ξ* represents the length-to-width ratio of the channel, *n* represents the axial compression ratio, and *λ* represents the shear span ratio.

**Figure 47:**
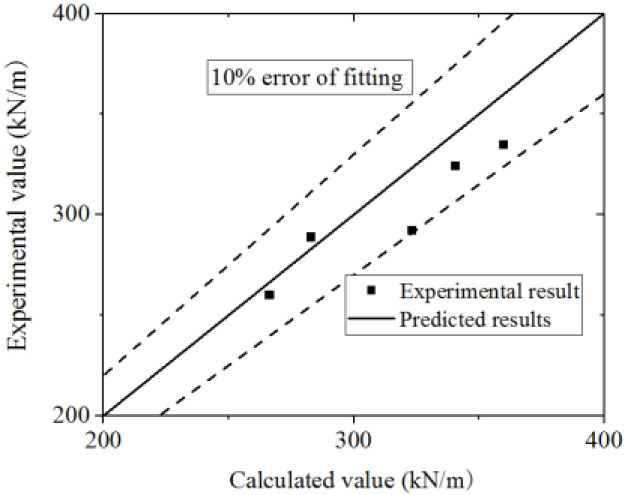
Comparison of elastic stiffness in the formula and test.

**Figure 48:**
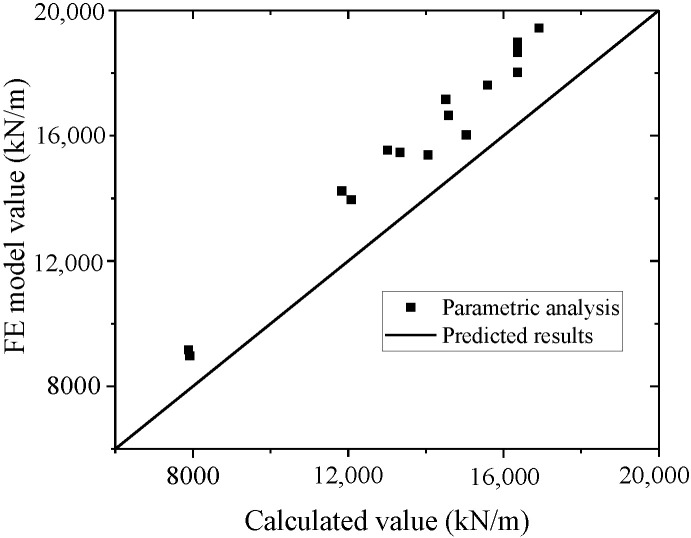
Comparison of elastic stiffness in the formula and parametric analysis.

A comparison of the test and parametric analysis results with the formula results, as shown in Figure 47 and Figure 48. The formula could fit the test with an error less than 10%. The values of the FE model are higher than those of the formula, which indicates that the formula could conservatively predict the FE model results. Thus, the formula could accurately predict the elastic stiffness of wall.

#### 5.2.4. Prediction of the Secant Stiffness of the Yield Point

The same method is used to calculate the secant stiffness of the yield point. The formula is proposed in Equations (24)–(27).

(24) $$E_{\mathrm{sc}}I_{\mathrm{sc}}=q_{m}(E_{s}I_{s}+0.4\cdot E_{c}I_{c})$$

(25) $$G_{\mathrm{sc}}A_{\mathrm{sc}}=q_{v}\cdot (G_{s}A_{s}+G_{c}A_{c})$$

(26) $$q_{m}=2.11+13.79\cdot \xi^{2}-7.85\cdot \xi +7.12\lambda^{2}-5.4\lambda$$

(27) $$q_{v}=-0.29-4.89\cdot \xi^{2}+3.20\cdot \xi -0.04\lambda^{2}-0.07\lambda$$

*q*_m_ = Flexural stiffness reduction coefficient.
*q*_v_ = Shear stiffness reduction coefficient.
*ξ* = Length-to-width ratio of the channel.
*λ* = Shear span ratio of the shear wall.

**Figure 49:**
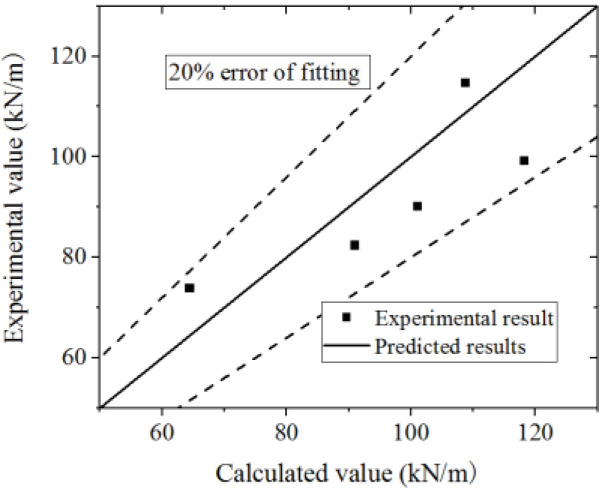
Comparison of secant stiffness of yield point in the formula and test.

**Figure 50:**
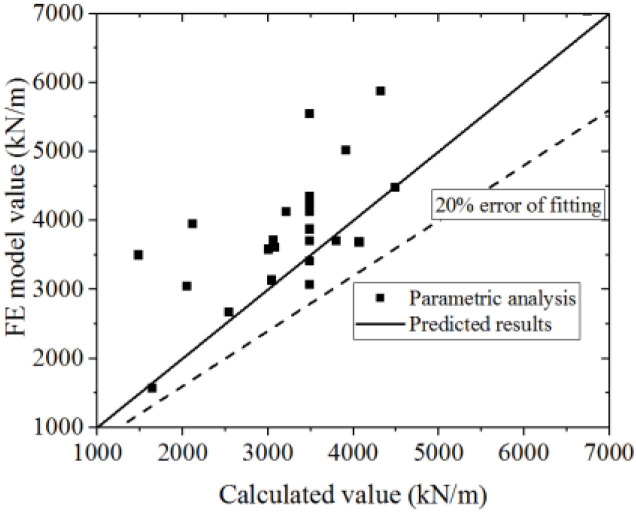
Comparison of secant stiffness of yield point in the formula and parametric analysis.

A comparison of the test and parametric analysis results with the formula results is shown in Figure 49 and Figure 50. The formula could fit the test with an error less than 20%. Most of the values of the FE model are higher than those of the formula, which indicates that the formula could conservatively predict the FE model results. The formula could accurately predict the secant stiffness of the yield point.

### 5.3. Comparison to Other Test Results

The formulas could accurately predict the ultimate strength capacity and stiffness of the test and parameter analysis accurately. To prove that the formula is universal for a similar composite shear wall, the formula is compared with the other results in the literature. Because the methods used for the stiffness of elastic and yield point are different, the elastic stiffness and secant stiffness of yield point are not compared. The comparison of the bearing capacities is shown in Table 2.

The errors between the formulas and tests in other studies are not more than 20%, indicating that the formula in this paper is also suitable for predicting the ultimate strength capacity and yield strength capacity of composite shear wall in different cross-section forms. Thus, the calculated formula has a certain universality.

## 6. Conclusions

In this paper, FE models are developed to predict the hysteresis behaviour of CWSC. The mechanical mechanism of the wall is studied. The effects of key design parameters on the bearing capacity and stiffness are investigated. The main conclusions could be drawn as follows:

1. The web plate and concrete are the main components that resisted the lateral force. The web plate is found to contribute to between 55% and 85% of the total shearing resistance of the wall.
2. The corner of wall mainly resisted the vertical force and the rest of wall resisted the shear force. The concrete is separated into several columns by the stiffened plates, each of which is independent and resisted by vertical force.
3. The elastic stiffness and ultimate strength capacity are enhanced with increasing wall thickness, steel ratio, axial compression ratio, and channel length-to-width ratio. The elastic stiffness and ultimate strength capacity are weakened with increasing shear span ratio.
4. The parameters affecting the ductility of CWSC are the steel ratio, shear span ratio, axial compression ratio and the length-to-width ratio of the channel. The influence of steel ratio and shear span ratio on ductility is positively correlated, while the axial compression ratio and the length-to-width ratio of the channel are negatively correlated.
5. Formulas are proposed to evaluate the ultimate strength capacity, yielding bearing capacity, elastic stiffness, and secant stiffness of the yield point of the composite shear wall. The formulas in this paper could predict the ultimate strength capacity more accurately than the formulas in specifications. Meanwhile, the formulas could predict the ultimate strength capacity in other tests from the literature well. The formulas can provide a basis for engineering design.

## Figures and Tables

**Table 1:** Summary of the samples.

Sample	Height of Wall (H/mm)	Cross Section of Wall (B × T/mm × mm)	Cross Section of Channel (b × T/mm × mm)
CWSC-1	1050	700 × 120	175 × 120
CWSC-2	1050	700 × 120	233 × 120
CWSC-3	1050	700 × 120	140 × 120
CWSC-4	1050	700 × 105	175 × 105
CWSC-5	1050	700 × 135	175 × 135

**Table 2:** Calculated and other test ultimate strength capacities and yielding bearing capacities.

Sample		Ultimate Strength Capacity			Yielding Strength Capacity		
		Test Value (kN)	Calculated Value (kN)	Error of Fitting	Test Value (kN)	Calculated Value (kN)	Error of Fitting
J.G. Nie [4] 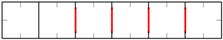	CFSCW-2	2539	2150	−15%	2153	1775	−18%
	CFSCW-4	2198	2005	−9%	1914	2005	5%
	CFSCW-5	2120	1906	−10%	1827	1906	4%
	CFSCW-6	2357	2098	−11%	1984	2098	6%
	CFSCW-10	1117	1052	−6%	954	874	−8%
	CFSCW-11	1365	1359	0%	1157	1142	−1%
	CFSCW-12	2018	1892	−6%	1748	1628	−7%
X.D. Ji [40] 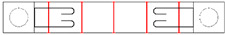	SW-1	814	865	6%	669	712	7%
	SW-2	809	856	6%	614	704	15%
	SW-3	669	700	5%	509	575	13%
	SW-4	799	774	−3%	597	636	7%
L.H. Chen [45] 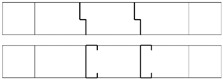	DSCW-L1	777	653	−16%	515	523	2%
	DSCW-L2	785	674	−14%	465	542	17%
	DSCW-L3	801	694	−13%	553	560	1%
	DSCW-C1	791	694	−12%	521	560	8%
L.H. Guo [17] 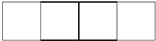	CSW-8	457	448	−2%	392	373	−5%
X. Zhang [11] 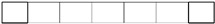	YZQ-1	999	932	−7%	776	755	−3%
	YZQ-2	947	1064	12%	758	873	15%
	YZQ-3	1288	1132	−12%	934	922	−1%
	YZQ-5	1209	1231	2%	953	1011	6%
	YZQ-7	2209	2075	−6%	1643	1710	4%

## Data Availability

The data presented in this study are available on request from the corresponding author. The data are not publicly available due to privacy.

## References

[1] Ramesh S. (2013). Behavior and Design of Earthquake-Resistant Dual-Plate Composite Shear Wall Systems. Ph.D. Thesis.

[2] Bruneau M., Alzeni Y., Fouché P. (2013). Seismic Behavior of Concrete-Filled Steel Sandwich Walls and Concrete-Filled Steel Tube Columns.

[3] Chen Y., Chen P. (2017). Structural design and research on the 1000m-high Skyscraper of CSCEC. Build. Struct..

[4] (2010). ASCE 7-10. Minimum Design Loads for Buildings and Other Structures.

[5] (2010). AISC 341-10. Seismic Provisions for Structural Steel Buildings.

[6] Nie J.G., Hu H.S. (2013). Experimental study on seismic behavior of high-strength concrete filled double-steel-plate composite walls. J. Constr. Steel Res..

[7] Mydin M. (2011). Structural performance of lightweight steel-foamed concrete-steel composite walling system under compression. Thin Wall Struct..

[8] Wright H. (1998). Axial and bending behavior of composite walls. J. Struct. Eng..

[9] Wang M.Z., Guo Y.L., Zhu J.S. (2020). Flexural buckling of axially loaded concrete-infilled double steel corrugated-plate walls with T-section. J. Constr. Steel Res..

[10] Nie X., Wang J.J., Tao M.X. (2019). Experimental study of flexural critical reinforced concrete filled composite plate shear walls. Eng. Struct..

[11] Zhang X., Qin Y., Chen Z. (2016). Experimental seismic behavior of innovative composite shear walls. J. Constr. Steel Res..

[12] Zhang W.Y., Wang K., Chen Y., Ding Y.K. (2019). Experimental study on the seismic behaviour of composite shear walls with stiffened steel plates and infilled concrete. Thin Wall Struct..

[13] Nguyen N.H., Whittaker A.S. (2017). Numerical modelling of steel-plate concrete composite shear walls. Eng. Struct..

[14] Epackachi S., Whittaker A.S., Varma A.H. (2015). Finite element modeling of steel-plate concrete composite wall piers. Eng. Struct..

[15] Rafiei S., Hossain K.M.A., Lachemi M. (2013). Finite element modeling of double skin profiled composite shear wall system under in-plane loadings. Eng. Struct..

[16] FWei F., Zheng Z.J., Yu J. (2019). Computational method for axial compression capacity of double steel-concrete composite shear walls with consideration of buckling. Eng. Mech..

[17] Guo L.H., Wang Y.H., Zhang S.M. (2018). Experimental study of rectangular multi-partition steel-concrete composite shear walls. Thin Wall Struct..

[18] Epackachi S., Nguyen N.H., Kurt E.G. An experimental study of the in-plane response of steel-concrete composite walls. Proceedings of the 22nd International Conference on Structural Mechanics in Reactor Technology (SMiRT 22).

[19] Epackachi S., Nguyen N.H. (2014). In-plane seismic behavior of rectangular steel-plate composite wall piers. J. Struct. Eng..

[20] Li X., Li X. (2017). Steel plates and concrete filled composite shear walls related nuclear structural engineering: Experimental study for out-of-plane cyclic loading. Nucl. Eng. Des..

[21] Huang Z., Liew J. (2016). Structural behaviour of steel-concrete-steel sandwich composite wall subjected to compression and end moment. Thin Wall Struct..

[22] Yan J.B., Liew J., Zhang M.H. (2015). Experimental and analytical study on ultimate strength behavior of steel-concrete-steel sandwich composite beam structures. Mater. Struct..

[23] Qin Y., Shu G.P. (2019). Compressive behavior of double skin composite wall with different plate thicknesses. J. Constr. Steel Res..

[24] Kurt E.G., Varma A.H., Booth P. (2016). In-Plane Behavior and Design of Rectangular SC Wall Piers without Boundary Elements. J. Struct. Eng..

[25] (2014). AISC N690-12. Specification for Safety-Related Steel Structures for Nuclear Facilities.

[26] (2008). ACI 318-08. Building Code Requirements for Structural Concrete and Commentary.

[27] (2015). JGJ/T 380-2015. Technical Specification for Steel Plate Shear Walls.

[28] (1997). JGJ 101-1996. Specification of Testing Methods for Earthquake Resistant Building.

[29] Wang J.F., Li B.B., Li J.C. (2017). Experimental and analytical investigation of semi-rigid CFST frames with external SCWPs. J. Constr. Steel Res..

[30] Wierzbicki T., Bao Y.B., Lee Y.W. (2005). Calibration and evaluation of seven fracture models. Int. J. Mech. Sci..

[31] Cook J.W.H. (1985). Fracture characteristics of three metals subjected to various strains, strain rates, temperatures and pressures. Eng. Fract. Mech..

[32] Yu H.L., Jeong D.Y. (2010). Application of a stress triaxiality dependent fracture criterion in the finite element analysis of unnotched Charpy specimens. Theor. Appl. Fract. Mec..

[33] Bao Y. (2003). Prediction of Ductile Crack Formation in Uncracked Bodies. Ph.D. Thesis.

[34] Zhou T.H., Wen C.L.I., Guan Y. (2014). Damage analysis of steel frames under cyclic load based on stress triaxiality. Eng. Mech..

[35] Han L.H. (2007). Concrete Infilled by Steel Tube Structure-Theory and Practice.

[36] Wang Z.Q., Yu Z.W. (2004). Concrete Damage Model Based on Energy Loss. J. Build. Mater..

[37] Tran C.T.N., Li B. (2016). Initial Stiffness of Reinforced Concrete Columns with Moderate Aspect Ratios.

[38] Park R., Thompson K.J. (1974). Behavior of Prestressed, Partially Prestressed, and Reinforced Concrete Interior Beam-Column Assemblied under Cyclic Loading: Test Results of Units 1 to 7.

[39] Wang K., Zhang W.Y., Ding Y.K., Chen Y. (2021). The analysis of axial compression ratio and shear-span ratio of shear wall in high-rise buildings. Institute of Structural Stability and Fatigue, China Steel Construction Society. Ind. Constr..

[40] Ji X.D., Jiang F.M., Qian J.R. (2013). Seismic behavior of steel tube-double steel plate-concrete composite walls: Experimental tests. J. Constr. Steel Res..

[41] Huang L., Lu Y.Q., Xu Z.P. (2012). Correctional recommendation of bearing capacity formula of RC compression-bending members. Eng. Mech..

[42] Sheet I.S., Gunasekaran U., MacRae G.A. (2013). Experimental investigation of CFT column to steel beam connections under cyclic loading. J. Constr. Steel Res..

[43] (2010). JGJ 3-2010. Technical Specification for Concrete Structures of Tall Building.

[44] (2016). AISC 341-16. Seismic Provisions for Structural Steel Buildings.

[45] Chen L.H., Wang S.Y. (2019). Seismic behavior of double-skin composite wall with L-shaped and C-shaped connectors. J. Constr. Steel Res..

